# Virtual Screening Combined with Enzymatic Assays to Guide the Discovery of Novel SIRT2 Inhibitors

**DOI:** 10.3390/ijms24119363

**Published:** 2023-05-27

**Authors:** Naomi Scarano, Elena Abbotto, Francesca Musumeci, Annalisa Salis, Chiara Brullo, Paola Fossa, Silvia Schenone, Santina Bruzzone, Elena Cichero

**Affiliations:** 1 Department of Pharmacy, Section of Medicinal Chemistry, School of Medical and Pharmaceutical Sciences, University of Genoa, Viale Benedetto XV, 3, 16132 Genoa, Italy; naomi.scarano@edu.unige.it (N.S.); francesca.musumeci@unige.it (F.M.); chiara.brullo@unige.it (C.B.); paola.fossa@unige.it (P.F.); silvia.schenone@unige.it (S.S.); 2 Department of Experimental Medicine, Section of Biochemistry, University of Genoa, Viale Benedetto XV 1, 16132 Genoa, Italy; elena.abbotto@unige.it (E.A.); annalisa.salis@unige.it (A.S.); santina.bruzzone@unige.it (S.B.); 3 IRCCS Ospedale Policlinico San Martino, 16132 Genova, Italy

**Keywords:** sirtuin, virtual screening, molecular docking, assays, inhibitor, SIRT2, drug design

## Abstract

Sirtuin isoform 2 (SIRT2) is one of the seven sirtuin isoforms present in humans, being classified as class III histone deacetylases (HDACs). Based on the high sequence similarity among SIRTs, the identification of isoform selective modulators represents a challenging task, especially for the high conservation observed in the catalytic site. Efforts in rationalizing selectivity based on key residues belonging to the SIRT2 enzyme were accompanied in 2015 by the publication of the first X-ray crystallographic structure of the potent and selective SIRT2 inhibitor SirReal2. The subsequent studies led to different experimental data regarding this protein in complex with further different chemo-types as SIRT2 inhibitors. Herein, we reported preliminary Structure-Based Virtual Screening (SBVS) studies using a commercially available library of compounds to identify novel scaffolds for the design of new SIRT2 inhibitors. Biochemical assays involving five selected compounds allowed us to highlight the most effective chemical features supporting the observed SIRT2 inhibitory ability. This information guided the following in silico evaluation and in vitro testing of further compounds from in-house libraries of pyrazolo-pyrimidine derivatives towards novel SIRT2 inhibitors (**1**–**5**). The final results indicated the effectiveness of this scaffold for the design of promising and selective SIRT2 inhibitors, featuring the highest inhibition among the tested compounds, and validating the applied strategy.

## 1. Introduction

Sirtuins (SIRTs) belong to the class III protein deacetylases (HDACs) and are highly conserved from bacteria to humans. HDACs are enzymes that catalyze the removal of acetyl groups from acetylated lysine residues of (non)histone proteins to counteract the histone acetyltransferase (HATs) action. Recent studies have proven that sirtuins not only deacetylate substrates but also catalyze different post-translational modulations involving demyristoylation and desuccinylation [1,2,3,4].

As class III HDACs, SIRTs require the co-substrate nicotinamide adenine dinucleotide (NAD^+^) to exert their catalytic activity. In mammals, there are seven sirtuins (SIRT1–7), each possessing different biological and specific functions in various cell compartments [5,6,7]. Indeed, SIRT1 is mainly nuclear, though shuttling into the cytoplasm, while SIRT2 houses in the cytoplasm, shuttling to the nucleus. SIRT3-5 are caged in mitochondria and SIRT6; SIRT7 are localized in the nucleus [8]. SIRT2′s substrates for deacetylation include tubulins, ATP-citrate lyase, phosphoenolpyruvate carboxykinase, lactate dehydrogenase, and glucose-6-phosphate dehydrogenase [6,9,10]. SIRT2 is expressed in various organs, including brain, ovary, esophagus, heart, liver, lung, testicles, thyroid, and spleen. Numerous studies have revealed that SIRT2 has a dual function in the formation of malignancies, acting as both a tumor promoter or suppressor, depending on the cancer type [8]. On this basis, the development of SIRT2 inhibitors arresting the proliferation of cancer cells is of therapeutic relevance [11,12,13]. In addition, SIRT2 inhibition is reported as beneficial in several nervous system disorders, such as Parkinson’s Disease (PD), Alzheimer’s disease (AD), Huntington’s disease (HD), depression, and ischemic stroke [14,15,16]. Recent data support the neuroprotective role played by SIRT2 inhibitors in cognitive impairment [17], suggesting that SIRT2 has a critical role in neurological diseases, being a potential therapeutic target for most of them [18].

Like the other SIRT isoforms, SIRT2 has a central catalytic domain of approximately 270 amino acids, where a Rossman fold and a smaller domain with the NAD^+^-binding module and a zinc-binding one (ZBD) create the enzymatic active site [18,19,20]. The SIRT2 binding pocket is situated between the two domains in a wide hydrophobic groove [21,22].

**Figure 1:**
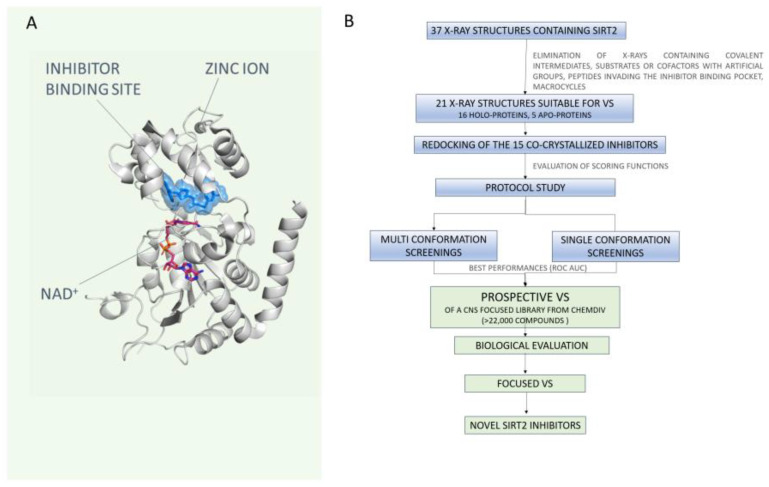
(**A**) X-ray crystallographic pose of SirReal2 at the SIRT2 binding site. (**B**) Workflow of the study. Thirty-seven X-rays are available on the PDB. Among them, unsuitable X-rays have been removed as described. A subset of 6 PDBs was used to perform a protocol study, allowing the selection of the best screening conditions. This information was used to perform initial single conformation VS with an annotated database. Biological evaluation and further optimization led to the identification of novel scaffolds featuring SIRT2 inhibitory ability.

Thus, the SIRT2 active site includes an acetyl-lysine channel and hydrophobic cavities in tandem with a ligand-induced selectivity pocket, as described for the well-known selective inhibitor SirReal2 [23]. A schematic representation of SirReal2 at the SIRT2 binding site is reported in Figure 1A.

Several published X-ray crystal structures of SIRT2 are available at the protein data bank [24,25], in different protein conformations [26,27,28], as well as in the presence or absence of the substrate or of enzyme inhibitors [29]. This large amount of data provides valuable information to pursue virtual screening (VS) campaigns toward new putative SIRT2-targeting compounds.

Herein, in the attempt to find novel scaffolds for the design of SIRT2 inhibitors as putative neuroprotective agents, we performed a Structure-Based Virtual Screening (SBVS) study using a ChemDIV library of more than 22,000 compounds [30] containing a set of molecules optimized in terms of pharmacokinetics (PK) for the Central Nervous System (CNS). The SBVS study refers to the 37 X-ray structures of SIRT2 present in the PDB. Among them, 16 crystals, including substrates or peptide inhibitors invading the binding pocket or artificial groups (e.g., trifluoro-) have been excluded. The remaining structures were exploited to develop a protocol study for setting the most appropriate conditions for the in silico screening. This has been achieved by taking into account different ligands and/or protein preparation conditions. The best-performing approach was maintained to preliminarily evaluate the ChemDIV library in terms of putative SIRT2 inhibitory ability. The workflow of the utilized in silico techniques combined with biological assays is shown in Figure 1B.

The biological in vitro results obtained for five selected ChemDIV compounds allowed us to point out the most effective chemical features turning in the desired SIRT2 inhibitory behavior. This information guided the following evaluation of further compounds from in-house libraries based on bioisostere and scaffold replacement approaches.

The final results obtained through the chosen in-house derivatives **1**–**5** confirmed the effectiveness of the pyrazolopyrimidine scaffold for the design of promising SIRT2 inhibitors also endowed with favorable PK properties.

## 2. Results

In this study, we employed a VS strategy combined with biological assays to unveil novel derivatives featuring SIRT2 inhibitory ability, relying on a consistent number of X-ray crystallographic data. After collecting the most representative data from the PDB, a protocol study was performed to identify the best conditions for the in silico screening.

Specifically, the effect of the ligand and/or protein preparation was evaluated through preliminary VS with an annotated database (2% of active compounds) and the calculation of the corresponding Receiver Operating Characteristic-Area Under the Curve (ROC-AUC) parameter. This approach was used to evaluate the screening performances of the collected SIRT2 X-ray structures, both with a single- and multi-protein conformation procedure. The best performing system was used to execute a preliminary VS involving the previously mentioned ChemDIV library. Moreover, the database compounds were conceived to maximize chemical diversity and were available for purchasing (and subsequent testing). The final VS results were analyzed according to a consensus approach among scoring functions (SFs). After visual inspection, five compounds were tested in vitro and then exploited to guide via scaffold replacement the following screening of in-house derivatives as promising SIRT2 inhibitors.

### 2.1. Virtual Screening Protocol Assessment

Several SIRT2 X-ray crystallographic data are available (37 PDB codes), many of them being related to protein-inhibitor complexes. Initially, all of them were collected before manually evaluating the most reliable for the following VS approach. Next, the PDB codes, including substrates or peptide inhibitors invading the binding pocket or artificial groups (e.g., trifluoro-), have been removed in order not to influence the ligand placement during the subsequent calculations (see Materials and Methods). Based on these criteria, we excluded 16 crystals among the 37 X-ray structures of SIRT2 present in the PDB. The retained 21 conformations are listed and detailed in Table 1.

**Figure 2:**
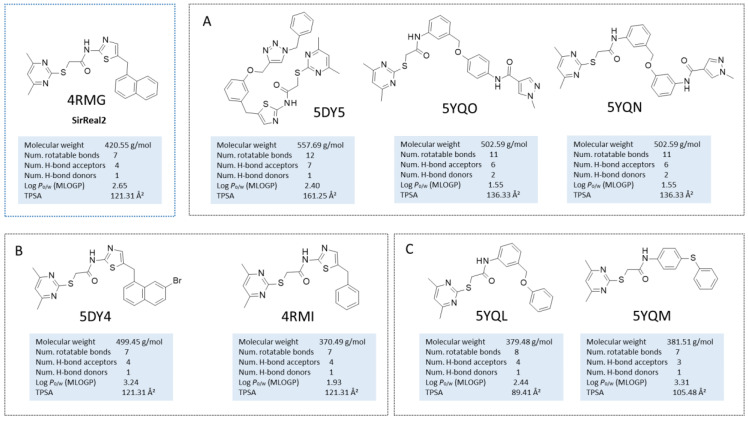
Chemical structure of the collected co-crystallized SIRT2 inhibitors as SirReal2 and related analogues. The reference compound is reported within the cyan box; the analogues developed via structural elongation of the prototype are shown in (**A**). The SirReal2 highly related analogues maintaining the thiazole core are shown in (**B**), whereas those bearing bioisostere replacement of the thiazole ring are reported in (**C**). Predicted druglikeness parameters have been reported, as obtained by SWISSADME (TPSA: topological polar surface area) [39].

Among the selected protein–ligand complexes, 15 out of 21 crystals contained a small molecule as a synthetic inhibitor, being nine out of these 15 co-crystals (4RMG, 5DY5, 5YQO, 5YQN, 5DY4, 4RMI, 5YQL, 5YQM, 4RMH) related to the well-known selective SIRT2 inhibitor SirReal2 and analogues (see Figure 2). 

Indeed, 4RMG and 4RMH also include SirReal2 (in the presence or absence of NAD^+^), whereas 5DY4 involves a highly related analogue featuring the *Br*-naphthyl group instead of the unsubstituted naphthyl one of SirReal2. Structural simplification of the same bicyclic ring alone, in the benzyl one, is shown in 4RMI. When this structural variation was applied with bioisostere replacement of the thiazole ring with a further phenyl ring, the co-crystallized analogues in 5YQL and 5YQM were obtained. Applying once again the mentioned bioisostere replacement of the main thiazole ring of SirReal2 in tandem with structural elongation at the related terminal aromatic ring led to the inhibitors in 5DY5, 5YQO, and 5YQN. To obtain more information regarding the collected PDB codes, the SIRT2 complexes involving SirReal2 and the analogues have been superposed and compared in silico via BLOSUM62 [40]. All eight SIRT2 co-crystals experienced highly comparable protein structure conformations, revealing a limited flexibility featured by the protein when highly related analogues are co-crystallized (see Figure S1A). This turns into very low values of RMSD on distances as estimated between each couple of proteins (see Figure S1B,C) with respect to the main protein structure (Main RMSD) or to the α-carbon atom CA (CA RMSD) comparison (mean value of the Main RMSD = 0.551 Å; the mean value of the CA RMSD = 0.542 Å). These crystals shared an identity percentage of at least 97%, suggesting a very limited number of not conserved residues or sequence gaps involving each couple of compared macromolecules (see Figure S1D).

**Figure 3:**
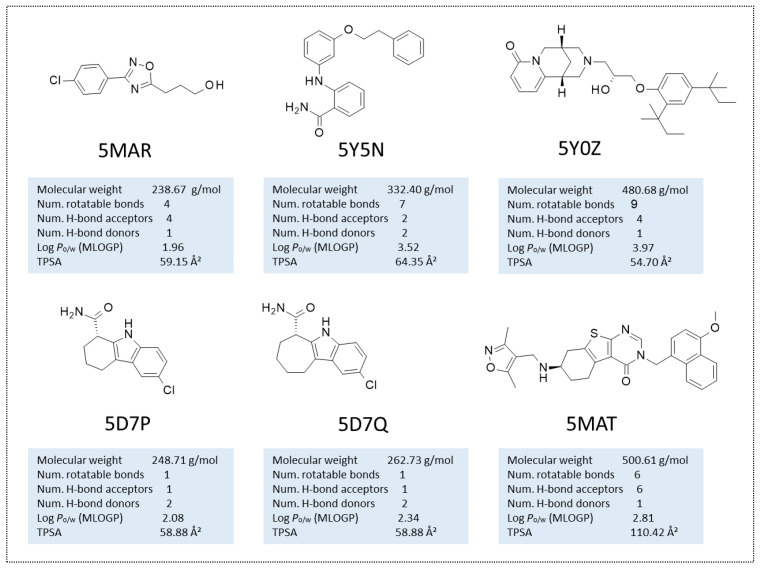
Chemical structure of the collected co-crystallized SIRT2 inhibitors featuring different chemo-types with respect to SirReal2. Predicted druglikeness parameters have been reported, as obtained by SWISSADME (TPSA: topological polar surface area) [39].

Non-sirReal2 compounds as SIRT2 inhibitors have been co-crystallized in the other six PDB codes, such as 5MAR, 5Y5N, 5Y0Z, 5D7P, 5D7Q, and 5MAT (see Figure 3).

Superimposition of these PDB codes indicated a higher flexibility of the SIRT2 protein in the presence of different bulky and/or flexible inhibitors, especially in the case of unconserved residues or sequence gaps between some couples of proteins if compared to the previous crystals containing, in any case, SirReal2 congeners (see Figure S2A,B).

**Figure 4:**
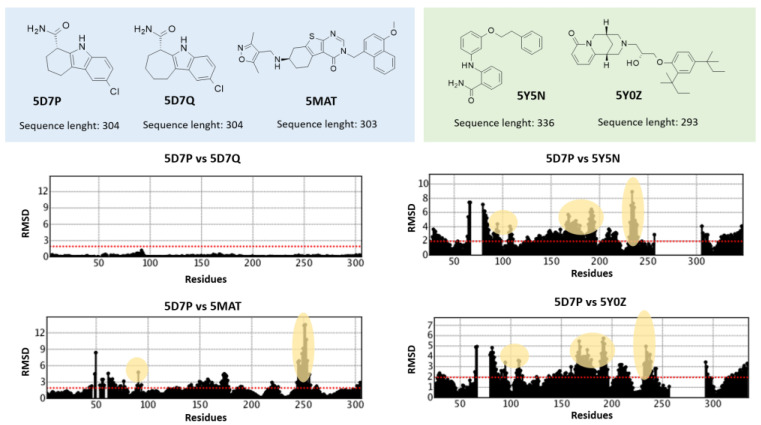
Schematic representation of the role played by differences in the inhibitor chemotype and/or in the protein sequence length to affect the SIRT2 conformation. RMSD trends, as obtained by superimposition of 5D7P, 5D7Q, 5MAT, 5Y5N, and 5Y0Z, are shown; the chemical structure of the co-crystallized inhibitors is also reported. Similar and dissimilar chemo-types are highlighted in light cyan and green. Residues belonging to the inhibitor binding site are highlighted in yellow. Gap in sequence length is shown in the RMSD plot.

As a result, the calculated RMSD values on distances between couples of these proteins (5MAR, 5Y5N, 5Y0Z, 5D7P, 5D7Q, and 5MAT) were higher than those previously obtained for the PDB codes involving SirReal2 congeners (see Figure S2A,B). Indeed, Main RMSD and CA RMSD were 1.993 Å and 2.003 Å, respectively, among the non SirReal2-like crystals. Among the six co-crystallized complexes, 5Y5N featured as a quite flexible and folded SIRT2 inhibitor and differed from the two very similar 5D7P, and 5D7Q, exhibiting a rather small, flat tricyclic-containing ligand. The PDB code 5Y0Z, including a bulky, branched SIRT2 inhibitor was also quite different from 5D7P, 5D7Q, and 5MAR, showing a small, rather flexible protein ligand. The effect played in terms of RMSD values by the most different chemo-types included within these PDB codes, in tandem with differences due to the crystal sequence length, is shown in Figure 4.

**Figure 5:**
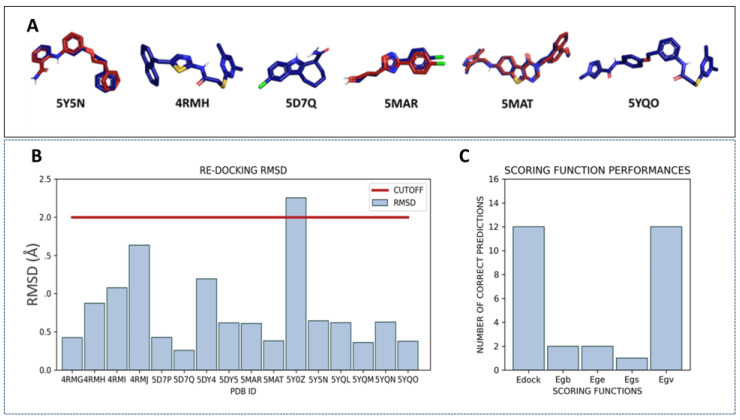
(**A**) Superposition of the docking pose (red) obtained by the re-docking of the crystallographic ligand with the crystallographic ligand itself (blue). The best poses identified by both the Edock and Egv are reported. One candidate for each chemotype is represented. (**B**) Re-docking performances. The plot reports the RMSD (Å) of the docking pose with respect to the corresponding crystallographic coordinates. (**C**) Scoring function performances. For each scoring function (Edock, Egb, Ege, Egs, and Egv) the number of cases in which the SF can recognize the lowest RMSD docking pose is reported.

On this basis, to preliminarily evaluate the most accurate docking protocol to be exploited for choosing the best-performing X-ray crystallographic data among the collected 16 PDB codes for VS, we performed a re-docking of the 16 crystallographic synthetic inhibitors. Examples of well re-docked ligands (RMSD value below 2 Å) are reported in Figure 5A (SIRT2 inhibitors belonging to the PDB codes 5Y5N, 4RMH, 5D7Q, 5MAR, 5MAT, and 5YQO are reported, as representative of the main chemotypes featured by the chosen 15 crystals).

Protein crystals whose co-crystallized inhibitor was predicted with an RMSD inferior to 2 Å were then taken into account as putative X-ray data to perform the following VS. As shown in Figure 5B, 15 ligands were correctly placed in the active site, whereas 5Y0Z ligand exhibited a RMSD higher than 2 Å. This conformation was excluded from further investigations. As 20 poses were produced for each re-docked ligand, it was possible to evaluate the performances of the scoring functions as the capability to rank first the lowest RMSD pose. The Egv and Edock scores were able to correctly select the best pose in 12/16 cases (Figure 5C), and they gave almost accurate results also in the remaining four cases.

These two scores represent an estimation of the van der Waals energy, and a global score, respectively. On this basis, Edock and Egv were considered for the following VS steps. 

Next, preliminary screenings with an annotated database were performed to select the more appropriate protocol identifying the most active compounds as best ranked with respect to the inactive ones. The resulting database included 510 compounds, with a ratio of 2% active compounds [41], reported in Table 2.

As mentioned above, the benchmarking database was used to screen a subset of six diverse inhibitor/SIRT2 complexes (5Y5N, 4RMH, 5D7Q, 5MAR, 5MAT, 5YQO), prepared according to different protocols. These X-rays were selected considering different chemotypes of inhibitors to take into account the subsequent differences in protein conformation. Performances were evaluated through the corresponding ROC-AUC, initially considering different protein preparation and ligand treatment approaches (see experimental section).

As shown in Figure S3, for most of the six enzyme-inhibitor complexes, the preparation protocol seems to be of secondary importance with respect to the choice of conformations. Thus, for subsequent calculations, we selected the H-optimization preparation level, which shows satisfying performances among the selected X-rays and the highest values for the best-performing X-ray of the subset (5Y5N).

**Figure 6:**
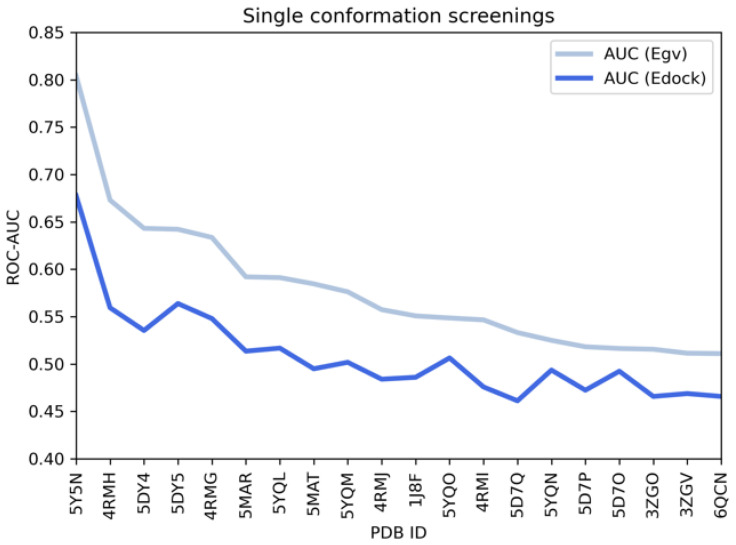
For each of the selected conformation (indicated by the corresponding PDB code) the ROC-AUC value was calculated. According to both the Egv and Edock scores, 5Y5N was identified as the best-performing structure. Moreover, the two scores were also in agreement regarding the selection of the top-five-performing conformations, used in the following multi-conformation step.

Single conformation screenings. To gain more information regarding the best-performing X-ray data for following VS, Benchmarking VS was performed, including the previously explored 15 crystals concerning SIRT2 synthetic inhibitors as well as apo-structures (1J8F, 3ZGV, 3ZGO, 5D7O, 6QCN). ROC-AUC was calculated, and the results are presented in Figure 6. 

Notably, the best performances were obtained using the 5Y5N crystal structure. 

Conversely, most of the apo-structures (1J8F, 3ZGV, 3ZGO, 5D7O, 6QCN) were properly ranked as less-performing. In the case of 5Y5N, the AUC was approximately 0.8 when calculated with the Egv and 0.68 with the Edock score. Moreover, both the considered scores were in agreement in the identification of the top five conformations (5Y5N, 4RMH, 5DY4, 5DY5, and 4RMG), which were selected for multi-conformation screenings. 

Multi-conformation screenings. The selected top five conformations were grouped to generate all the possible combinations and submitted to multi-conformation VS with the benchmarking database. The corresponding ROC-AUC was calculated and compared to the ones obtained by the single conformation approach (Figure S4). In multi-conformation screenings, the hitlist reports the score of the ligands docked in the most “convenient” protein conformation [47]. None of the multi-conformation screenings resulted in better performances than the best single conformation one (5Y5N). Thus, the 5Y5N single conformation was selected as the best-performing structural information.

Moreover, an additional multi-conformation screening was performed considering the other four X-rays (1J8F, 5Y5N, 5DY5, 5D7O) exhibiting good performing ability in active-to-inactive compounds ranking, thanks to different scoring functions (Edock-Egv, Egs, Ege, Egb, Table S1).

The corresponding ROC-AUC was 0.59 for the Edock score and 0.64 for the Egv. 

Compared with the single conformation screenings (ROC-AUC = 0.80), this type of approach did not outperform the best single conformation screening.

### 2.2. Prospective Virtual Screening of SIRT2 Inhibitors

**Figure 7:**
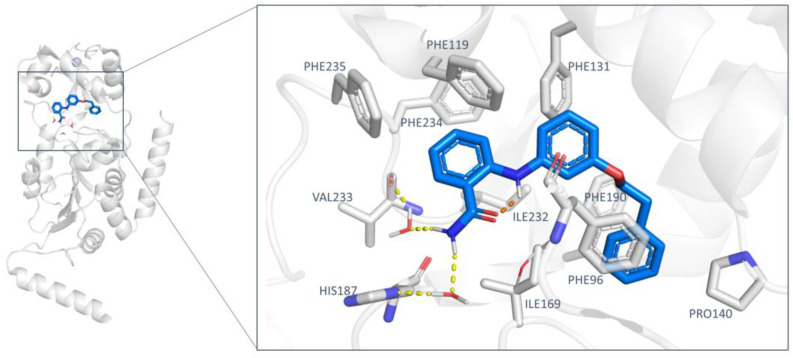
The 5Y5N co-crystallized ligand (binding pose). H-bonds are represented as dashed yellow lines.

The ChemDIV database was therefore screened against the 5Y5N crystal structure with a single conformation approach. Using the mentioned CNS-focused library would allow screening a relatively small library while maintaining a certain diversity among compounds, enabling to explore diversified scaffolds at a reduced computational cost. Moreover, the included compounds were predicted to exhibit BBB permeability and optimized druglike properties. The reference X-ray structure (5Y5N) is a co-crystal containing a selective nicotinamide derivative, which occupies the active site at the interface of the Zinc binding domain and the Rossmann fold. This ligand is able to induce the formation of the selectivity pocket. In particular, the phenoxyethylphenyl moiety establishes π–π and H–π interactions with the surrounding residues (Phe131, Leu134, Leu138, Tyr139, Pro140, Phe143, and Ile169). Moreover, the 2-aminobenzamide moiety is involved in water-mediated hydrogen bonds with His187 (side chain) and the main chain of Val233. An intramolecular H-bond between the amide and the aromatic amine confers additional rigidity to the ligand (Figure 7) [34].

**Figure 8:**
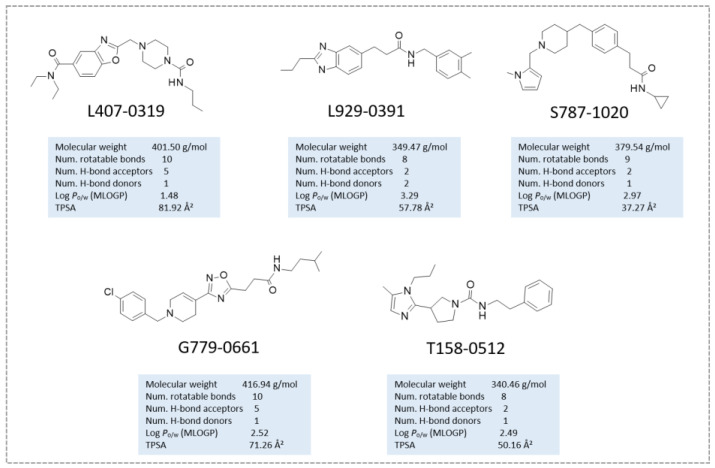
Chemical structure of the selected ChemDiv derivatives via preliminary VS, as putative SIRT2 inhibitors. Predicted druglikeness parameters, as obtained by SWISSADME, have been reported (TPSA: topological polar surface area) [39].

After the screening, a number of candidates for the following in vitro tests were identified. The choice was performed by taking into consideration the consensus among the Egv and the Edock scoring functions. In particular, the VS hitlist was duplicated, and the two obtained lists were ordered according to the Egv or Edock. The top 1000 compounds of the two lists were compared, and the common compounds were retained, obtaining a final VS hitlist of 144 candidates (see Table S2). The corresponding docking poses were visually inspected and superimposed to the 5Y5N co-crystal. Ligands exhibiting a similar position compared to the crystallographic ligands were prioritized. Indeed, convenient putative interactions were considered to partially simulate the co-crystallized interaction mode. In the molecule selection process, care was devoted to exploring diverse scaffolds to be exploited as putative new SIRT2 inhibitors, leading to five different compounds, namely, **L407-0319**, **T158-0512**, **L929-0391**, **S787-1020,** and **G779-0661** (Figure 8). 

The binding poses of these preliminary chosen compounds are shown in Figure S5. Among them, **L407-0319** was able to maintain polar contacts with Val233 as the 5Y5N co-crystallized ligand, moving the benzoxazole ring towards the reference inhibitor phenoxy group (Figure S5D). A comparable docking mode was also experienced by the benzimidazole ring and the disubstituted phenyl ring of **L929-0391**, respectively (Figure S5C). Conversely, removing every heterocyclic central ring led to weak positioning, relying especially on Van der Waals contacts with the surrounding residues, as featured by **S787-1020** and **T158-0512** (Figure S5E,F).

To assess the reliability of the obtained docking poses, subsequent molecular docking studies were performed via MOE2019.01 software [48] applying the Induce Fit method, which allows moving the binding site flexible chain during calculations (see Table S3 for the scoring function and top ranked positioning). This approach was expected to better investigate the putative docking mode of the retained five compounds.

**Figure 9:**
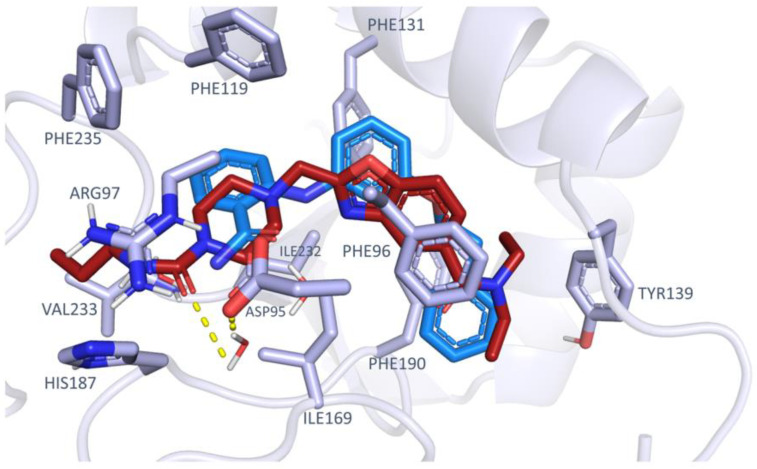
Docking pose of **L407-0319** (C atom; dark orange) as obtained via Induced Fit method, in comparison with that of the 5Y5N co-crystallized SIRT2 inhibitor (C atom; blue). H-bonds are shown as yellow dotted lines.

According to our calculations, no relevant changes in the predicted binding mode were observed for **T158-0512**, **L929-0391**, and **S787-1020** (see Figure S6), supporting a maintained polar contact involving Val233. Conversely, compound **G779-0661** showed a reversed docking positioning compared to the previous one, obtained by preliminary VS, being H-bonded to Val233 as the previous derivatives (compare Figure S6 to Figure S5B). This also allowed the compound to move the p-*Cl*-benzyl group in proximity to the alkoxy-phenyl portion of the 5Y5N co-crystallized ligand. **L407-0319** experienced a different docking positioning when evaluated via the Induced Fit method, highlighting water-mediated H-bonds between the oxygen atom of the carbonyl group tethered to the piperazine substituent and Asp95, Gln167 (Figure 9). This positioning guarantees π−π stacking involving the benzoxazole ring and Phe96, detecting Van der Waals contacts between the terminal alkyl chains and Ala135, Phe190, and Val233.

The biological data revealed that three out of five ChemDIV library ligands featured a modest SIRT2 inhibitory ability, the compound **L407-0319** being the most interesting (see the following section for biochemical assays). 

Notably, the effectiveness of SIRT2 inhibitors featured by the selected ChemDiv compounds in tandem with their different chemical structure, in terms of main scaffold and related substituents, provided complementary information with those already available in the literature. This data allowed us to proceed further with the in silico evaluation of in-house derivatives as novel SIRT2 inhibitors, described as follows. 

### 2.3. Focused VS of Pyrazolopyrimidines as SIRT2 Inhibitors

Based on the results obtained via biochemical assays involving the chosen ChemDIV compounds, the introduction of a central bicyclic heteroaromatic ring allowed to exhibit SIRT2 inhibitor ability, as shown by **L407-0319** and **L407-0319**. This main scaffold should be accompanied by two hydrophobic terminal groups, with one of them being an aromatic ring or an H-bonding substituent. Taking into account the previously described X-ray data for SIRT2, 85% of the collected SIRT2-inhibitor complexes featured two aromatic terminal groups (see previous Figure 2 and Figure 3), with at least one of them being a heteroaromatic ring or endowed with H-bonding moieties. This information motivated further in silico screening to identify novel SIRT2 inhibitors by focusing on pyrazolo-pyrimidine derivatives as the main chemotype.

Specifically, compounds **1**–**5** were explored to partially fulfill the requirements derived from our previous VS campaign. Synthesis of compounds **1**, **3**–**5** [49] and compound **2** [50] has already been reported by us in the search for antitumor agents in terms of *trans* isomers.

**Figure 10:**
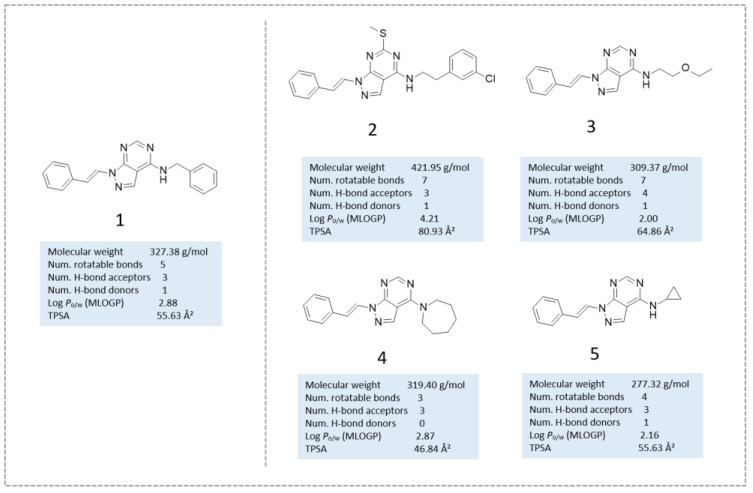
Scheme of the chemical structure of the pyrazolo-pyrimidine derivatives **1**–**5**, investigated as putative SIRT2 inhibitors. Predicted druglikeness parameters, as obtained by SWISSADME, have been reported (TPSA: topological polar surface area) [39].

As shown in Figure 10, compound **1** represents the reference compound of this series, bearing two terminal unsubstituted phenyl rings tethered to the central pyrazolo-pyrimidine core, while the analogues **2**–**5** allowed to evaluate the putative effectiveness of further aromatic or non-aromatic groups, featuring H-bonding moieties.

In silico evaluation of these derivatives has been performed via molecular docking studies by MOE2019.01 software [48], applying the Induce Fit method, as mentioned, to refine docking studies for the ChemDiv compounds. To ascertain the reliability of this docking protocol to identify promising SIRT2-targeting compounds, the known SIRT2 inhibitor **AGK2** [51] was also submitted to molecular docking calculations. Table S4 reports the scoring function values and related top ranked positioning for **1**–**5** and **AGK2**. Regarding the corresponding best-ranked docking poses, **AGK2** proved to be highly superposed to the bioactive positioning featured by the 5Y5N ligand (see Figure S7). 

**Figure 11:**
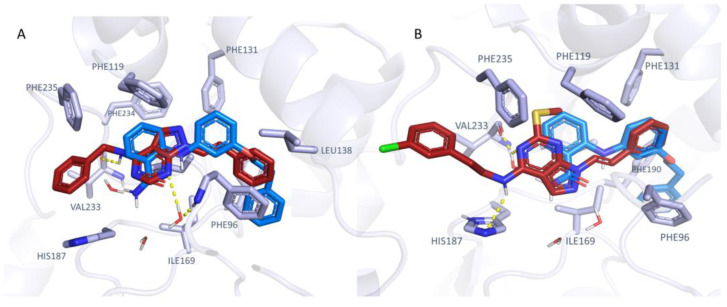
Docking pose of 1 (C atom; dark orange) (**A**) and of 2 (**B**) as obtained via induced fit method, in comparison with that of the 5Y5N co-crystallized SIRT2 inhibitor (C atom; dark blue). H-bonds are shown as yellow dotted lines.

According to our calculations, the pyrazolo-pyrimidine core of **1**–**5** mimicked the amide-substituted aniline portion of the 5Y5N co-crystallized inhibitor. In detail, the choice of two aromatic features as terminal pendants linked to the main bicyclic ring was highly encouraged, as shown by **1**–**2**, as well as the introduction of flexible H-bonding groups in place of one of the mentioned phenyl rings, as in **3**. As shown in Figure 11, the **1**–**2** phenyl-alkyl amine group was projected towards Phe235 and His187, detecting π−π stacking and cation-π contacts, being also (either directly or water-bridged mediated) H-bonded to Val233.

Based on this substituent length, the inhibitor was also stabilized at the enzyme crevice via additional water-mediated and direct H-bonds with Phe96 (inhibitor **1**) and His187 (inhibitor **2**). This positioning allowed the vinyl moiety of the two compounds **1**, **2** to simulate the phenoxy group and the aniline portion of the 5Y5N co-crystallized inhibitor, respectively. As a result, the compound **2** vinyl-substituted phenyl ring was superposed on the phenoxy group of the reference inhibitor. This docking mode was also featured by analogue **3**, taken as representative of the pyrazolo-pyrimidine series displaying an alkyl-containing terminal group in place of an aromatic ring. This structure guaranteed contact with His187, Val233, and Phe96 (see Figure S8). Conversely, the choice of bulky H-bonding features instead of hydrophobic and planar ones, such as the two phenyl rings of **1**–**2**, was predicted as detrimental, impairing the inhibitor positioning in the proximity of Val233. 

Next, biochemical assays were performed to assess the putative SIRT2 inhibitor ability experienced by the pyrazolopyrimidine derivatives and to evaluate the reliability of the whole computational study. As shown in the following section, the compound **1**–**5** inhibitory ability increases from compound **1** to **5**, being in good accordance with the lower scoring function (S) values of the best ranked **1**–**2** (S = −10.1235 to −10.6011 kJ/mol) when compared to **3**–**5** (−8.9098 to −10.2455 kJ/mol), as shown in Table S4. Then, the best ranked pose of the most potent inhibitor **AGK2** was endowed with the lowest S value (S = −13.3422 kJ/mol) as the most effective SIRT2 inhibitor explored herein.

### 2.4. Biochemical Assays

An HPLC-based assay was used to determine the effect of the selected compounds on SIRT2 deacetylase activity upon incubation of recombinant SIRT2 with an acetylated peptide (H3K9Ac, a peptide acetylated on Lys 9) and NAD^+^. The percentage of SIRT2 activity inhibition obtained with each compound at a 150 μM concentration is reported in Table 3. In parallel, the inhibition exerted by **AGK2** at the same concentration was estimated at 97 ± 10%. 

The selectivity of the most promising structure (compound **1**) for SIRT2 against other sirtuins was also determined. Compound **1** inhibition on the deacetylase activity catalyzed by SIRT1 and SIRT3 was 76 and 33%, respectively. In addition, the pyrazolopyrimidine prototype **1** did not affect SIRT6 deacetylase nor depalmitoylase activity; the latter was determined using a palmitoylated peptide (H3K9Palm). Thus, the identified compound is selective over SIRT6 and SIRT3 but not over SIRT-1.

### 2.5. In Silico Prediction of ADMET Properties

To accelerate the drug discovery process, in silico prediction of absorption, distribution, metabolism, excretion, and toxicity (ADMET) properties is regarded as a valuable supporting tool [52,53]. In searching for novel promising SIRT2 inhibitors, we evaluated herein several druglikeness properties for compounds **1**–**5**. The results have been compared with those calculated for reference inhibitor SirReal2. 

Taking into account the well-known Veber’s [54] and Lipinski’s rules [55], prediction of the (i) logarithmic ratio of the octanol-water partitioning coefficient (cLogP), (ii) molecular weight (MW) of derivatives, (iii) H-bonding acceptor number (HBA), or H-bonding donor groups (HBD), (iv) number of rotatable bonds (nRot_bond) and (v) topological polar surface area (TPSA), has been performed (Table S5). 

All the new compounds and the reference compound SirReal2 fulfill Lipinski’s and Veber’s rules.

In addition, the prediction of ADME parameters was also developed in terms of human intestinal absorption (HIA), estimation of the plasmatic protein binding event (% PPB), the volume of distribution (Vd), ligand affinity toward human serum albumin (LogKa has), and putative oral bioavailability, as a percentage (F %) (see Table S5). 

Next, in silico evaluation of toxicity properties was taken into account, including prediction of the probability of human ether-a-go-go related gene (hERG) channel inhibition at clinically relevant concentrations (Ki < 10 μM) (Table S6), and of cytochrome inhibition and lethal dose via mouse oral administration. Endocrine system disruption events and PAINS (Pan Assay Interference structures) were also estimated (Table S6). Notably, the newly developed compounds **1**–**5** were endowed with low toxicity, based on (hERG) channel inhibition or endocrine system disruption events, and with high values of the predicted LD_50_ descriptor (Table S6).

Current in silico profiling models or websites predicting potential molecule liabilities, such as off-target adverse drug reactions (ADRs), are deeply exploited to sustain the drug development process [56,57]. To further support the druglike profile of the newly identified SIRT2 inhibitors **1**–**5**, we proceeded to predict putative biological target(s) for this series of compounds via SwissTarget [58].

As shown in Figure S9, both the two pyrazolopyrimidines **1**–**2**, taken as representative promising SIRT2 inhibitors were predicted to be kinase-targeting ligands, classified as enzyme binders (the probability of being SIRTs- and kinases- targeting compounds spanning between 13–26% and 26–33%, respectively). This information is in accordance with previous biological assays regarding the pyrazolo-pyrimidine role as kinase inhibitors [49,50]. 

Similar behavior was also predicted for the analogues **3**–**5**, with probability values of interacting with SIRTs- and kinases- ranging between 13–20% and 20–46%, respectively. The prediction of affinity towards other protein families was less significant, anticipating limited off-target effects caused by compounds **1**–**5**.

Similarly, SirReal2 was also recognized as a putative kinase and enzyme-targeting ligand, with an estimated probability of binding to kinases of approximately 46%. 

## 3. Discussion

The developed workflow was aimed at maximizing the precision and performance of the VS campaign, with particular care for the choice of structural information. OC-AUC measurement to indicate screening performances was previously used to evaluate X-ray structures’ suitability [59] and ensemble of conformations’ performances [60]. Nevertheless, this approach is inevitably bias-affected due to scoring functions as classical pitfalls. Indeed, in different studies described in the literature, preliminary VS studies have been accompanied by subsequent molecular dynamic simulations (MD) to assess the stability of the derived protein–ligand poses [61].

Herein, we tried to make every choice based on the consensus among two scoring functions. The study outputs indicated 5Y5N as the best structure to perform an SBVS under the conditions used. In addition to the presented results, this choice shows good agreement with ROC analysis reported by Djokivic et al. [62], which identified the same conformation (together with 5MAT) among the most-performing for an SBVS. Moreover, this conformation exhibits the formation of the so-called “selectivity pocket”. This additional sub-pocket is created upon the binding of some classes of ligands and is thought to be responsible for ligand selectivity [22]. The presence of this extra volume increases the probability of including selective inhibitors. The study led to the identification of three compounds with very weak (**G779-0661**, **L929-0391**) and weak (**L407-0319**) activity over the five tested. A limited activity of compounds as a result of VS is expected [63], as this technique is not intended to produce ready-made drug candidates, but to provide new hints for the development of novel compounds, e.g., individuating novel scaffolds to be optimized. For this reason, compounds were tested for their activity on the enzyme at a concentration in the micromolar range, not to exclude possible candidate scaffolds to be optimized. In silico improvements may be obtained, for example, by considering more scoring functions and different software. Additionally, the inspected chemical space was rather limited. The used BBB library, indeed, contained only 22,000 compounds, in which chemical moieties were often repeated, although with different topologies. Herein, oxadiazole, benzoxazole, and indole-like scaffolds were reported in relation to SIRT2 inhibition [5,64], giving useful features to further screening novel chemotypes acting as SIRT2 inhibitors. By combining the structural information suggested by the assayed ChemDiv compounds with those already featured by the collected co-crystallized SIRT2 inhibitors, we were able to proceed with in silico screening and subsequent biochemical assays of a small library of in-house pyrazolo-pyrimidine derivatives [1,2,3,4,5]. As a consequence, the applied VS strategy could represent a useful approach to set up the identification of novel chemo-types targeting further proteins such as phosphodiesterase or enzymes, whose structure has been widely defined in the literature. The final results of our studies pointed out the effectiveness of the pyrazolo-pyrimidine scaffold for developing novel and selective SIRT2 inhibitors, with **1**–**5** featuring the most promising inhibition ability among the evaluated derivatives from ChemDiv and the in-house library.

## 4. Materials and Methods

### 4.1. Computational Studies

All the studied compounds were manually built by the MOE2019.01 Builder program and then parametrized (AM1 partial charges as calculation method), and energy was minimized by the Energy Minimize Program using MMFF94x forcefield of MOE2019.01 and RMS (root mean square) gradient equal to 0.0001, the root mean square gradient being the norm of the gradient times the square root of the number of (unfixed) atoms. This allowed to produce a single low-energy conformation for each ligand [48].

All the selected X-ray data of SIRT2 in the presence of different inhibitors or other compounds were collected from the Protein Data Bank [65].

#### 4.1.1. Manual Selection of the Protein Conformations and Redocking Calculations

Among the 37 PDBs containing the structure of SIRT2, only 21 were taken into account for the study. The exclusion criteria were the following:

1. Presence of substrates or peptidic inhibitors invading the binding pocket. The inclusion of these structures would prevent the positioning of the ligand in the active site, whereas their exclusion would generate a construct leaving an “empty” space which is peptide-induced, and therefore not suitable for the identification of small molecule-like inhibitors
2. Presence of (peptidic) substrates with artificial groups (e.g., Trifluoro-) for the same reason described, featuring in addition artificial groups.
3. Structures containing Carba-NAD. Despite the fact that it may seem a small modification, the O/C substitution is near to the binding pocket, and may influence the ligand placement.

The set of retained conformations has been reported in Table 1.

The 16 co-crystals included in the presented set were submitted to a re-docking step. The structures of the ligands were manually drawn using the MOE2019.01 builder tool. Ligands were then minimized (RMS = 0.00001), and partial charges were calculated according to the AM1 method implemented in MOE2019.01 [48,66]. The corresponding protein was prepared in ICM-Pro v9.2-c by Molsoft [67,68], the selected software for docking; VS. Waters and uninteresting ligands were delated, and a hydrogen-optimization step was performed prior to the removal of the ligand. The binding pocket was defined around the co-crystallized inhibitor. The docking effort (thoroughness) was set to 2. Twenty poses were generated for each of the re-docked ligand. The adherence of the obtained pose to the crystallographic position of the compound was evaluated through the RMSD value, calculated with the “kept in place” option available in ICM-Pro. 

#### 4.1.2. Benchmarking Database Creation

To evaluate the capability of a conformation to enrich a subset of active compounds with respect to a large number of decoys, preliminary screenings with a benchmarking database were performed. The Receiver Operating Characteristic-Area Under the Curve (ROC-AUC) parameter was selected to estimate the screening performance. The benchmarking database was built as follows:

Active compounds selection: A set of active molecules was collected by the analysis of data from the literature (starting from a recent review on the available SIRT2 inhibitors [64]; among these compounds, a subset of 10 small molecules was selected, following a maximum diversity approach, similarly to [69] Ligands with MW over 1000 or unspecified stereocenters were removed. Compounds with pronounced activities were prioritized. No ligand with % of inhibition lower than 99% or IC_50_ above 5 μM was retained. 

Decoy generation: The Directory of Useful Decoys-Enhanced (DUD-E) [69] automatic tool was chosen to generate decoys on the basis of the selected active molecules. Briefly, for each active ligand, a set of 50 decoys was generated, possessing similar physico-chemical properties, but dissimilar 2D topology [70]. The obtained database of 510 compounds (2% active compounds ratio) was used as benchmarking database to establish the protocol and to evaluate the performances of single- and multi-conformation VS.

#### 4.1.3. VS Protocol Study

Screenings of the benchmarking database on six conformationally diverse SIRT2 protein structure (5Y5N, 4RMH, 5D7Q, 5MAR, 5MAR, 5YQO) were performed, chosen within the previously retained 22 structures. Four levels of preparation for the protein (namely, Pnp, PHopt, PHisGln, Pmin), and three for the ligand (Lnp, Lpt, Lfull) were studied.

The meaning of the preparation protocols is the following: (i) Pnp: addition of hydrogens, (ii) PHopt: the assignation of H was performed to optimize the hydrogen bond network, (iii) PHisGln: some of the residues were allowed to change. His: three protonation state were explored; Asn, Gln: allowed to rotate of an angle of 180 degreed; Cys: if, near to metals, cysteins were treated differently (cym); (iv) Pmin: after H optimization, the protein was minimized following the Cartesian method implemented in ICM Pro.

For the ligands: (i) Lnp: ligands were represented as smiles and the 2D structures were generated, (ii) Lpt: molecules were assigned the correct tautomer and protomer (prevalent form at pH = 7.4) by means of the “wash” function implemented in MOE2019.01, (iii) Lfull: the predominant protomer in its predominant tautomeric state was generated, and the obtained structures were submitted to energy minimization and partial charges assignation with the AM1 method implemented in MOE2019.01. The ligand preparation protocol was studied with the PHopt level of preparation of the protein.

The VSs were performed with a thoroughness of 2.00, maintaining the protein and compound structures as rigid and flexible, respectively. The considered scoring functions were the Egv (grid-based Van der Waals energy) and the Edock (a global scoring function).

The removal of the ligand was performed after the minimization step. The protein preparation protocol was studied with the “washed” database of ligands as described above.

#### 4.1.4. VS via Single Conformation Study

The 20 protein conformations which survived the re-docking step were submitted to benchmarking VS with the previously described database and preparation protocol (see Section 2.1 Virtual screening protocol assessment). Single conformation screenings were performed at a thoroughness of 2. In the case of apo-structures, a superposition with an inhibitor-bound X-ray (5Y5N) was performed, and the binding pocket was identified around the 5Y5N ligand. ROC-AUC were calculated according to Egv and Edock scores. Top five conformations were selected for the multi-conformation study.

#### 4.1.5. VS via Multi-Conformation Study

The same method was applied to investigate the effect of considering multiple conformations in the VS. The top five X-ray structures obtained from the single conformation study were grouped in the 26 possible combinations (10 combinations of 2, 10 combinations of 3, 5 combinations of 4, and 1 combination of 5). Proteins were uploaded in ICM Molsoft and converted to ICM object as previously described. The structures were superimposed to each other, and a stack of conformation was built. The binding pocket was defined around a selection comprising all the present ligands. The docking thoroughness was of 6.7 for double, 10 for triple, 13,3 for quadruple, and 16.7 for quintuple conformation VS.

The prospective virtual screening was carried out with MolSoft ICM Pro v9.2-c, using the single conformation docking procedure. A BBB-focused ChemDIV library of 22,790 compounds was downloaded (https://www.chemdiv.com/catalog/focused-and-targeted-libraries/cns-bbb-library/, accessed on 28 April 2023) and prepared assigning the correct protomer and tautomer with the MOE2019.01 “wash” function. The obtained database was submitted to screening against the 5Y5N crystal structure. The pocket was identified around the co-crystallized ligand, and the screening was run at a thoroughness of 2.00. After calculation, the hitlist was duplicated, and the two lists were ordered according to Edock and Egv, respectively. The top 1000 compounds from both lists were compared, and common compounds were taken into account: a set of 144 compounds was selected and visually inspected; five candidates were chosen for in vitro tests.

#### 4.1.6. Induced Fit Docking

To assess the reliability of the obtained docking poses via preliminary VS of the ChemDiv libraries, as well as to develop a focused VS on the in-house pyrazolopyrimidine derivatives, molecular docking studies via Induced Fit method were performed (MOE2019.01 software) [48]. This approach allows to consider the protein binding site as flexible during calculations.

As regards the DOCK tool implemented in MOE2019.01, the template similarity methodology was applied, choosing as binding site the one occupied by 5Y5N co-crystallized inhibitor, including all those residues placed at 4.5 Å distance from the aforementioned inhibitor. The software tool works by placing ligands in the enzyme cavity based on the selected reference inhibitor. During calculation, template and input compounds are aligned via an undirected heavy-atom and projected feature triplet matching scheme. The applied scoring function is related to reference/ligand similarity terms as well as a protein–ligand clash term.

Calculation of the enthalpy-based Affinity dG scoring function allowed to score the obtained fifty poses, whereas the Induced Fit method refined the previous poses to the final ten docking poses, maintaining the Affinity dG as definitive scoring function for the final pose ranking.

The Induced Fit approach allows to maintain flexible protein sidechains within the selected binding site which are to be evaluated in the refinement stage. The derived docking conformers were prioritized by the score values of the lowest energy pose of the derivatives docked to the enzyme structure, as follows: S: the final score (corresponding to affinity dG), which is the score of the last stage of refinement, E_place: score from the placement stage; E_score1 and E_score2 score from rescoring stages 1 and 2; E_refine: score from the refinement stage, calculated to be the sum of the van der Waals electrostatics and solvation energies, under the Generalized Born solvation model (GB/VI).

Details of the applied docking protocol are described in our previous studies [71].

### 4.2. Enzymatic Assays

#### 4.2.1. H3K9Ac and H3K9Palm Peptide Synthesis

Peptides H3K9Ac and H3K9palm were synthesized using the standard 9-fluorenylmethoxycarbonyl (Fmoc) strategy of solid-phase peptide synthesis, as previously described [71]. The final products were judged to have a purity of 95% or higher, based on analytical HPLC/MS analysis. After lyophilisation, the peptides were stored as solid powders at −80 °C. Alternatively, they were dissolved in DMSO and stored at −20 °C.

#### 4.2.2. Evaluation of SIRT2, SIRT1 and SIRT3 Deacetylase Activity

Recombinant human SIRT1 (CAT# 10011190) and SIRT2 (CAT# 10011191) were supplied from Cayman Chemical; recombinant human SIRT3 (CAT# SRP0117) was obtained from Sigma-Aldrich S.r.l, Milan, Italy. 

In a 30 µL reaction phosphate buffer, SIRT1, SIRT2, or SIRT3 (at 17, 270 or 200 nM final concentration, respectively) were incubated with peptide H3K9Ac (240 µM), NAD^+^ (100 µM for SIRT2 and SIRT3; 500 µM for SIRT1) and in the presence or absence of the different compounds to be tested (or with the addition of 1 µL DMSO, control) at 37 °C. Compounds were tested at 150 µM final concentration. The reaction was stopped after 10 min by adding 3 volumes of an acidic methanol solution (200 mM HCl and 320 mM CH3COOH). After removal of the protein by centrifugation, aliquots of the supernatants were subjected to HPLC analysis as in [72], with slight modifications (Agilent Technologies 1260 HPLC, ZORBAX^®^ Eclipse Plus C18 3.5 μM, 4.6 mm × 10 mm column and flow set at 1 mL/min). Solvents used were of analytical grade.

#### 4.2.3. Evaluation of SIRT6 Deacetylase and Depalmitoylase Activity

Recombinant SIRT6 was synthesized as previously reported [73]. The purity of the recombinant protein was confirmed by electrophoresis. The obtained pellet was then resuspended in 20% glycerol (final concentration of the stock protein was 68.8 µM).

To assay the depalmitoylase activity, recombinant SIRT6 (4.6 µM) was incubated with a palmitoylated peptide (H3K9Palm, 100 µM), NAD^+^ (100 µM) and the different compounds to be tested (or with 1 µL DMSO, control) at 37 °C for 30 min. The reaction was carried out in 30 µL of a 20 mM Tris 7.4 pH buffer, containing 4 mM MgCl_2_. The deacetylase activity was assayed in the presence of a known activator (compound MDL-800), being SIRT6 endowed with a weak deacetylase activity. Compounds were tested at 150 µM final concentration. Optimal separation of substrate and product was obtained with two different HPLC methods for H3K9Ac/H3K9 and H3K9Palm/H3K9. For H3K9Ac/H3K9, the mobile phase consists of eluent A and B, with the following gradient of eluent B: 0–5 min 5%; 5–16 min, 5–25.1%; 16–20 min, 100%; 20–24 min, 100%; 24–24.1 min, 100–5%, at 1 mL/min. Eluent A and B were H_2_O + Trifluoroacetic acid 0.05%, and Acetonitrile + Trifluoroacetic acid 0.02%, respectively. For H3K9Palm/H3K9, the mobile phase consists of eluent A and B, with the following gradient of eluent B: 0–5 min 5%; 5–29 min, 5–49%; 29–35 min, 49–100%; 35–40 min, 100%; 40–40.1 min, 100–5%, at 1 mL/min. Eluent A and B were H_2_O + Formic Acid 0.1%, and Acetonitrile + Formic Acid 0.1%, respectively. HPLC system and column were as above.

### 4.3. In Silico Prediction of ADMET Properties

The prediction of all the reported ADMET parameters was developed by means of the Advanced Chemistry Development (ACD) Percepta platform [74,75]. The software prediction is performed based on the software implemented training libraries, which include experimentally determined pharmacokinetic and safety properties for different series of compounds. Prediction of PAINS (Pan Assay Interference structures) and of the derivative putative biological target(s) were derived via SwissADME website [76] and SwissTarget [58], respectively.

## Figures and Tables

**Table 1:** Retained SIRT2 X-ray structures after exclusion of unsuitable protein–ligand complexes.

Entry	PDB ID	Inhibitor	Cofactor	Substrate	Resolution (Å)	Ref.	Sequence Length	Year
1	1J8F	None	None	None	1.70	[20]	323	2001
2	3ZGV	None	ADPR	None	2.27	[31]	325	2013
3	3ZGO	none	none	None	1.63	[31]	325	2013
4	4RMG	SirReal2	NAD	None	1.88	[22]	304	2015
5	4RMH	SirReal2	None	Ac-Lys-H3 peptide	1.42	[22]	304	2015
6	4RMI	SirReal1	None	Ac-Lys-OTC peptide	1.45	[22]	304	2015
7	4RMJ	Nicotinamide	ADPR	none	1.87	[22]	304	2015
8	5D7P	EX-243	ADPR	None	1.76	[32]	304	2015
9	5D7O	none	ADPR	None	1.63	[32]	310	2015
10	5D7Q	CHIC35	ADPR	None	2.01	[32]	304	2015
11	5DY4	SirReal analog	NAD	None	1.77	[33]	304	2016
12	5DY5	SirReal probe	None	None	1.95	[2]	304	2016
13	5Y5N	Small molecule-inhibitor	None	None	2.30	[34]	336	2017
14	5MAR	oxadiazole	ADPR	None	1.89	[35]	303	2017
15	5MAT	Thienopyrimidinone	None	None	2.07	[35]	303	2017
16	5YQL	A2I	None	None	1.60	[36]	306	2018
17	5YQM	A29	None	None	1.74	[36]	306	2018
18	5YQN	L55	None	None	1.60	[36]	306	2018
19	5YQO	L5C	None	None	1.48	[36]	306	2018
20	5Y0Z	NPD11033	None	None	2.00	[37]	293	2018
21	6QCN	Quercetin (out of the protein)	ADPR	None	2.23	[38]	304	2019

**Table 2:** List of the ten active compounds selected for the benchmarking database generation.

Molecule	Activity (IC_50_, %inhib)	SMILE Notation	References
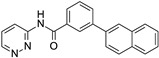	0.90 μM	O=C(Nc1cccnn1)c2cccc(c2)c3ccc4ccccc4c3	[42]
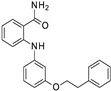	1.74 μM	NC(=O)c1ccccc1Nc2cccc(OCCc3ccccc3)c2	[34]
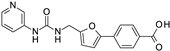	99%	OC(=O)c1ccc(cc1)c2oc(CNC(=O)Nc3cccnc3)cc2	[43]
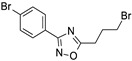	1.5 μM	BrCCCc1onc(n1)c2ccc(Br)cc2	[35]
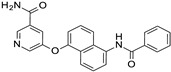	0.0483 μM	NC(=O)c1cncc(Oc2cccc3c(NC(=O)c4ccccc4)cccc23)c1	[44]
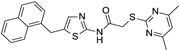 SirReal2	0.4 μM	Cc1cc(C)nc(SCC(=O)Nc2ncc(Cc3cccc4ccccc34)s2)n1	[22]
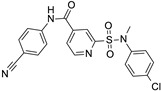	4.9 μM	CN(c1ccc(Cl)cc1)S(=O)(=O)c2cc(ccn2)C(=O)Nc3ccc(cc3)C#N	[45]
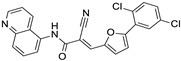 3-AGK2	1.56 μM	Clc1ccc(Cl)c(c1)c2oc(\C=C(/C#N)\C(=O)Nc3cccc4ncccc34)cc2	[44]
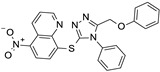	3.5 μM	[O-][N+](=O)c1ccc(Sc2nnc(COc3ccccc3)n2c4ccccc4)c5ncccc15	[46]
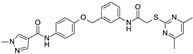	0.815 μM	Cc1cc(C)nc(SCC(=O)Nc2cccc(COc3ccc(NC(=O)c4cnn(C)c4)cc3)c2)n1	[36]

**Table 3:** Inhibition of SIRT2 enzymatic activity. Each compound was tested at the final concentration of 150 μM.

Molecule	SIRT2 Activity, %Inhibition (at 150 μM)
**L407-0319**	44.3 ± 5.3
**L929-0391**	24.4 ± 6.2
**S787-1020**	NI
**T158-0512**	NI
**G779-0661**	25.2 ± 5.9
**1**	81.2 ± 7.3
**2**	79.9 ± 5.7
**3**	51.1 ± 15.2
**4**	35.7 ± 2.5
**5**	31.4±
**AGK2**	97 ± 10

NI = No Inhibition.

## Data Availability

No data available.

## Supplementary Materials

The supporting information can be downloaded at: https://www.mdpi.com/article/10.3390/ijms24119363/s1.
