# Supplementary material for: Virtual Screening Combined with Enzymatic Assays to Guide the Discovery of Novel SIRT2 Inhibitors
Source: Int J Mol Sci. 2023 May 27;24(11):9363. doi: 10.3390/ijms24119363 (PMC10253774; doi:10.3390/ijms24119363)
Supplement: Supplementary file 1 [file ijms-24-09363-s001.zip › ijms-2399812-supplementary.pdf]

SUPPORTING INFORMATION

Naomi Scarano [1], Elena Abbotto [2], Francesca Musumeci[1], Annalisa Salis[2], Chiara Brullo[1], Paola Fossa[1], Silvia Schenone[1], Santina Bruzzone[2,3], Elena Cichero[1*]

[1] Department of Pharmacy, Section of Medicinal Chemistry, School of Medical and Pharmaceutical Sciences, University of Genoa, Viale Benedetto XV, 3, 16132 Genoa, Italy; naomi.scarano@edu.unige.it (N.S.); francesca.musumeci@unige.it (F.M.); chiara.brullo@unige.it (C.B.); paola.fossa@unige.it (P.F.); silvia.schenone@unige.it (S.S.)
[2] Department of Experimental Medicine, Section of Biochemistry, University of Genoa, Viale Benedetto XV 1, 16132 Genoa, Italy; elena.abbotto@unige.it (E.A.); annalisa.salis@unige.it (A.S.); santina.bruzzone@unige.it (S.B.)
[3] IRCCS Ospedale Policlinico San Martino, Genova, Italy

* Correspondence: elena.cichero@unige.it (E.C.); Tel.: +39-010-353-8350 (E.C.)

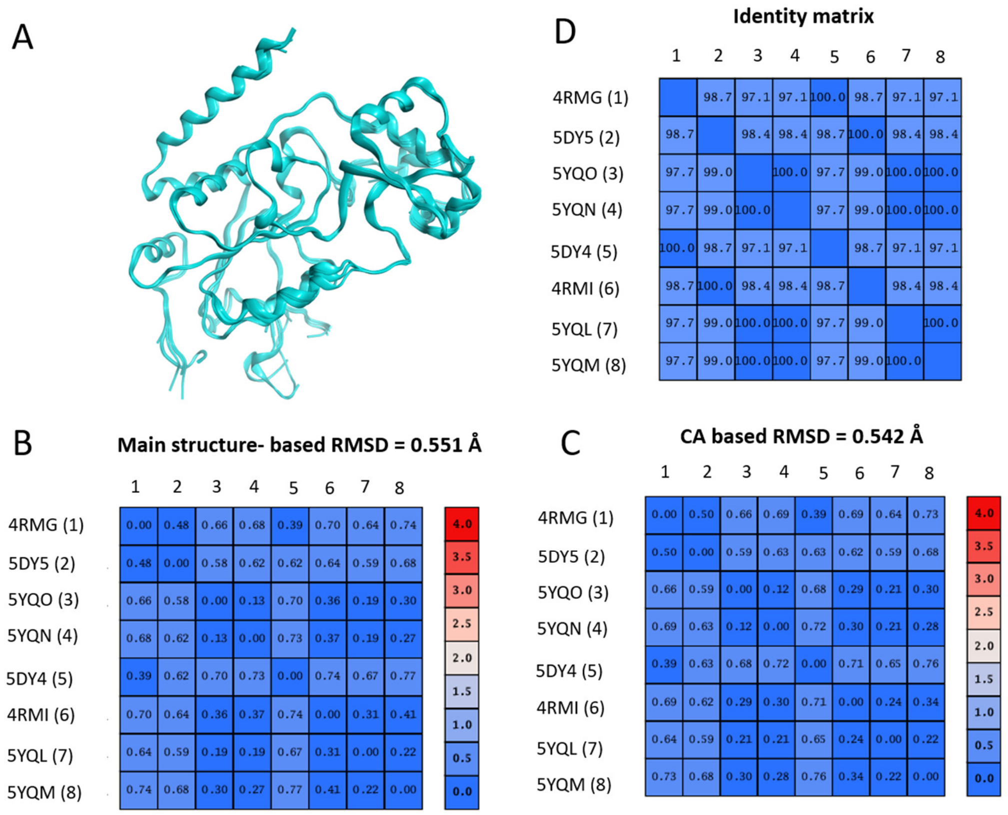

Figure S1. Superimposition of the SIRT2 X-ray complexes 4RMG, 5DY5, 5YQO, 5YQN, 5DY4, 4RMI, 5YQL, 5YQM, featuring SirReal2 and related analogues as enzyme inhibitors (A). Scheme of the derived RMSD matrix based on distances as estimated between each couple of proteins with respect to the main protein structure (B) or to the alpha-carbon atom CA (C) comparison. The related identity matrix is also shown (D).

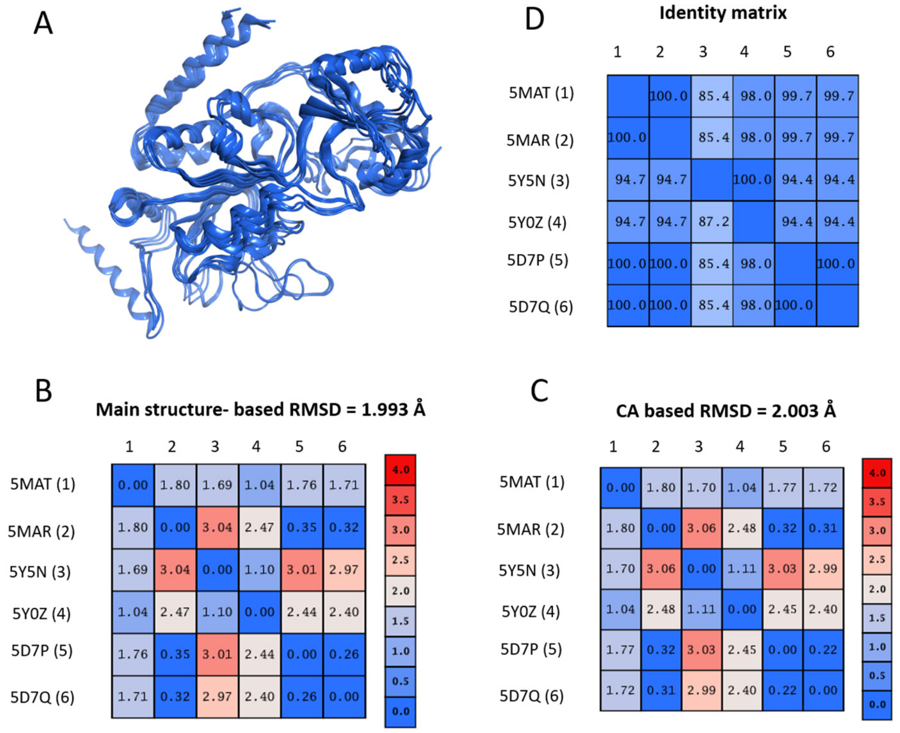

Figure S2. Superimposition of the SIRT2 X-ray complexes 5MAT, 5MAR, 5Y5N, 5Y0Z, 5D7P, 5D7Q, featuring non-SirReal2 chemical structure (A). Scheme of the derived RMSD matrix based on distances as estimated between each couple of proteins with respect to the main protein structure (B) or to the alpha-carbon atom CA (C) comparison. The related identity matrix is also shown (D).

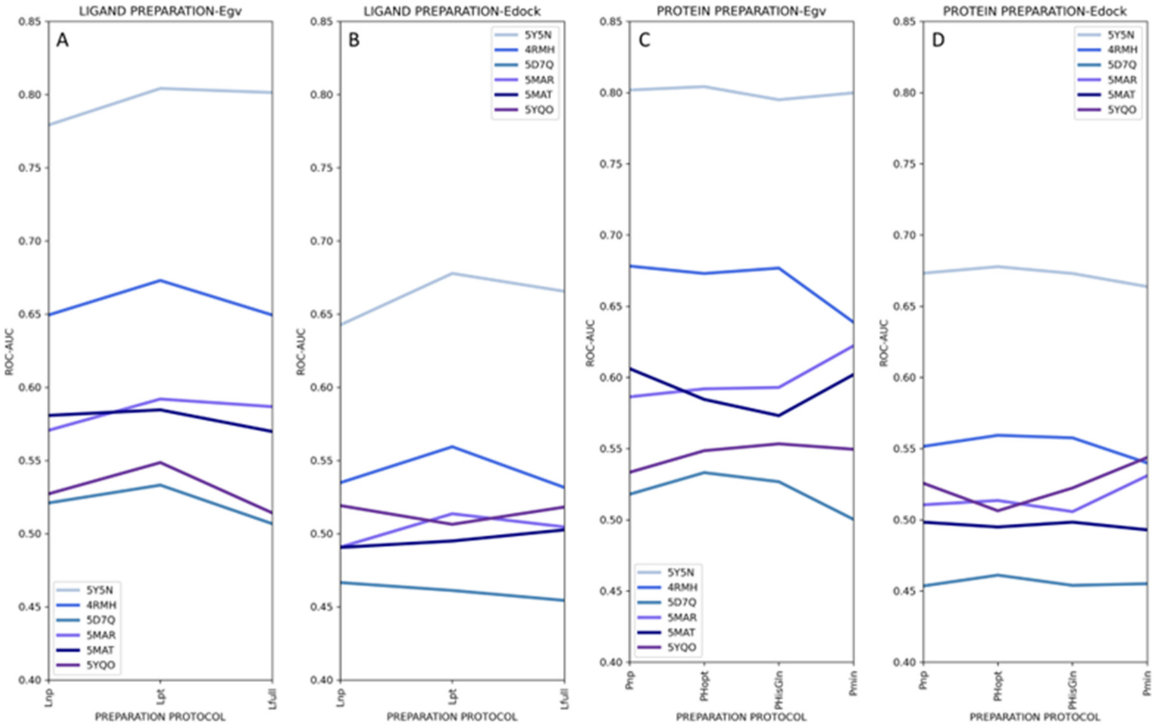

Preparation modality (L)	Description
Lnp	Ligands were submitted as smiles and 2D structures were generated
Lpt	Assignation of the correct protomer and tautomer
Lfull	Additional minimization and partial charges assignation

Preparation modality (P)	Description
Pnp	H were added with no optimization
PHopt	H were added and optimized to obtain convenient interactions
PHisGln	His, Pro, Asn, Gln, Cys were allowed to flip to obtain convenient interactions
Pmin	Assignation of partial charges and minimization

Figure S3. Preparation protocol effect on screening performances for a subset of conformationally diverse X-Rays (5Y5N, 4RMH, 5DYQ, 5MAR, 5MAT, 5YQO). Both the ligand (A-B) and the protein (C-D) preparation was investigated, according to the two selected scoring functions E-gv and E-dock.

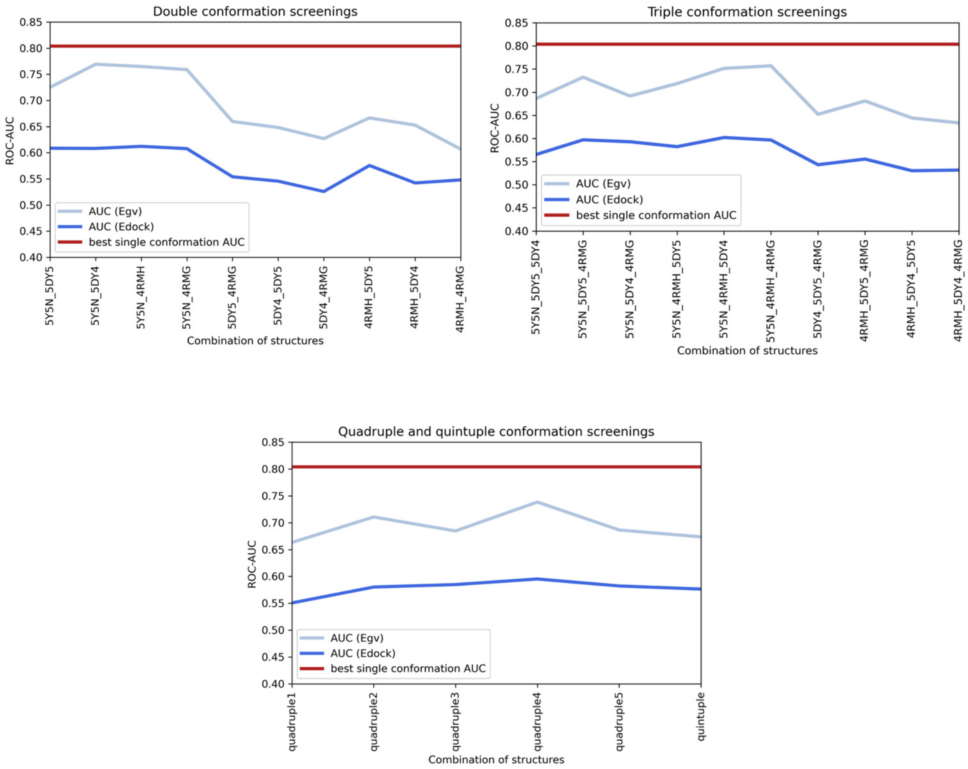

Figure S4. Multi-conformation screening performances. The AUC values of the screenings are reported on the y-axis, for each ensemble of conformation (x-axis). No multi-conformation screening outperformed the best single conformation AUC (shown as red line).

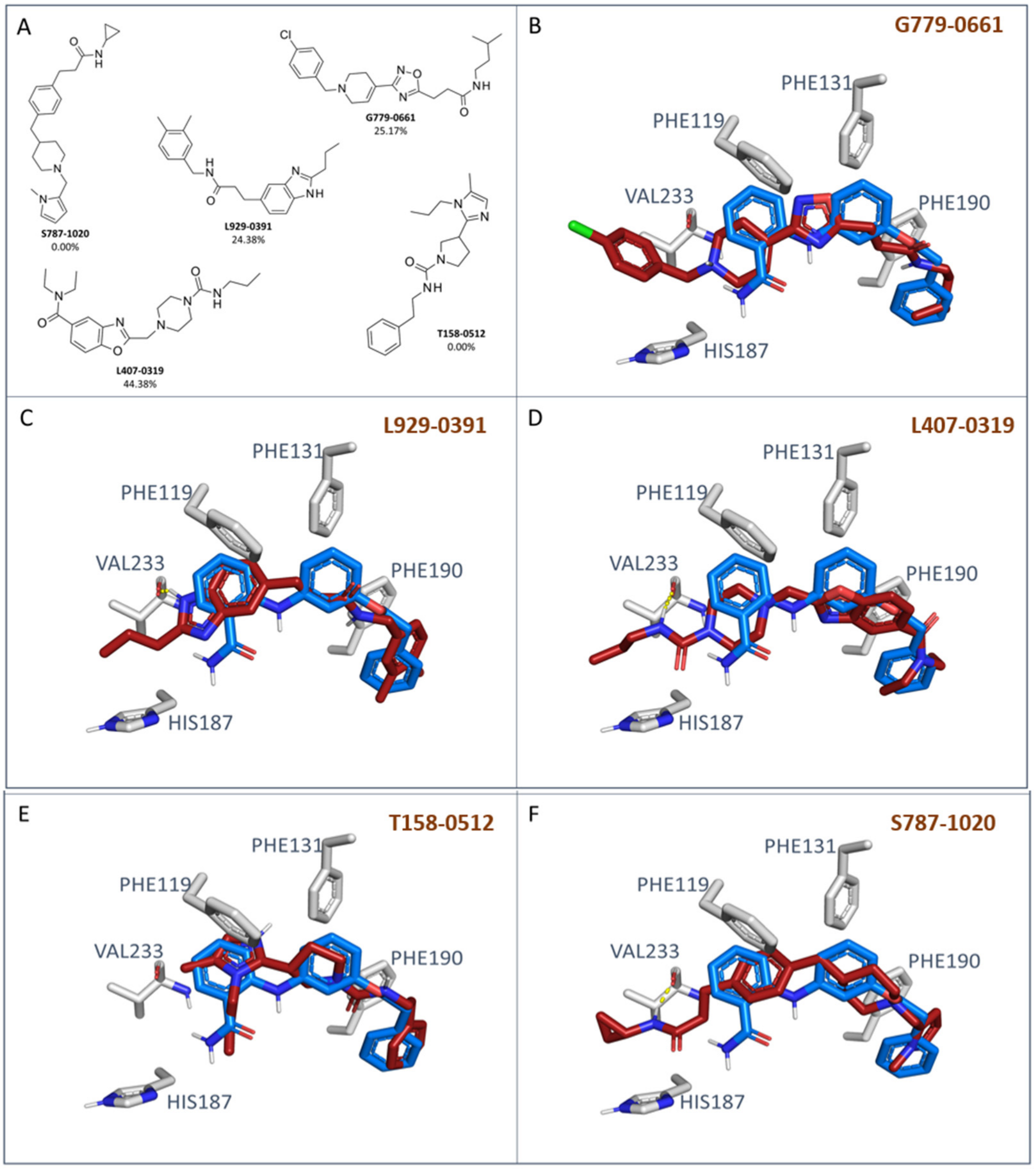

Figure S5. Tested compounds structures and their percentage of inhibition (@150microM) (A). Putative binding mode (docking pose) as obtained via preliminary ICM-Pro docking studies about: the (weakly) active compounds (dark red), in comparison with the crystallographic ligand of 5Y5N (blue) (B-D); inactive compounds (E-F). H-bonds are shown as yellow dotted lines.

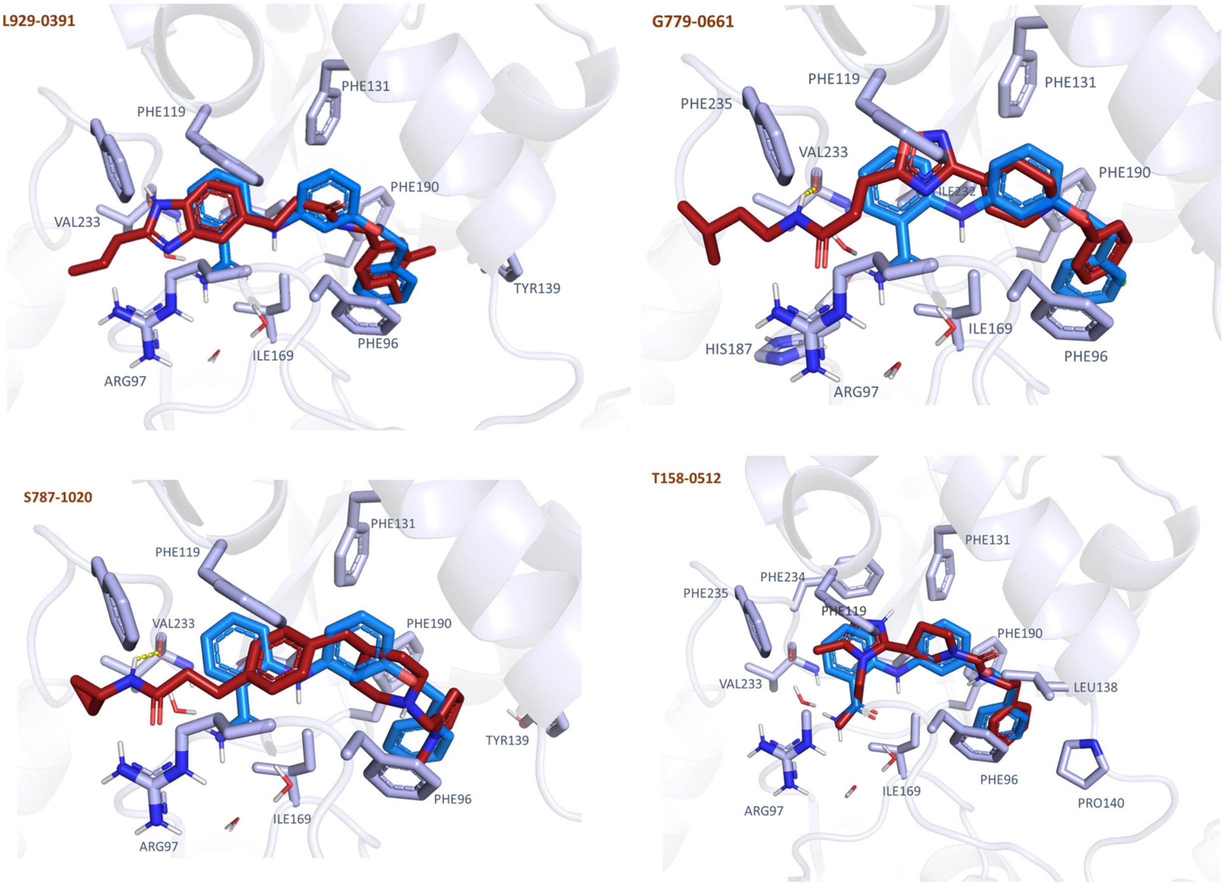

Figure S6. Molecular docking poses of the selected ChemDiv compounds T158-0512, L929-0391, S787-1020 and G779-0661 in dark red, as derived via Induced Fit method, are shown. The X-ray crystallographic positioning of the 5Y5N inhibitor is also reported (blue). H-bonds are shown as yellow dotted lines.

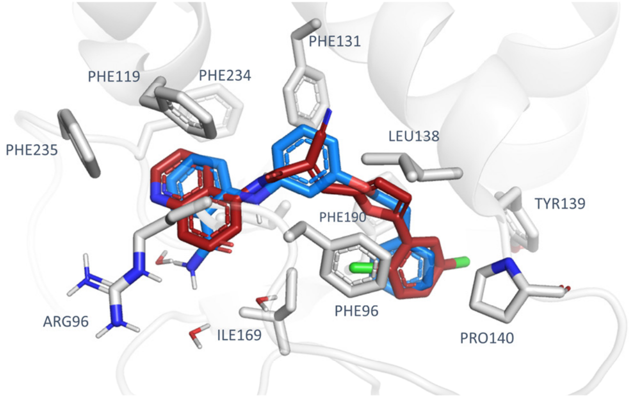

Figure S7. Molecular docking poses of **AGK2** in dark red, as derived via Induced Fit method. The X-ray crystallographic positioning of the 5Y5N inhibitor is also reported (blue).

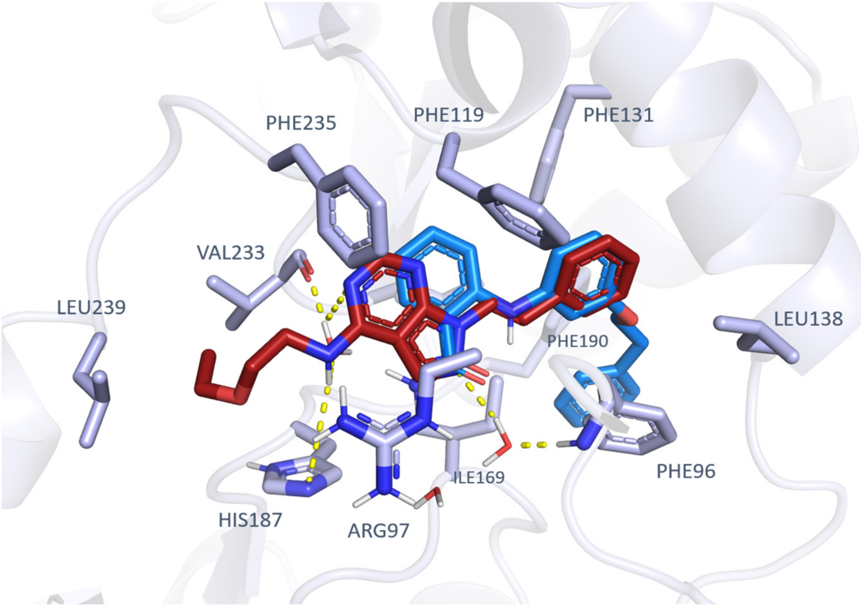

Figure S8. Molecular docking poses of compound **3** in dark red, as derived via Induced Fit method. The X-ray crystallographic positioning of the 5Y5N inhibitor is also reported (blue). H-bonds are shown as yellow dotted lines.

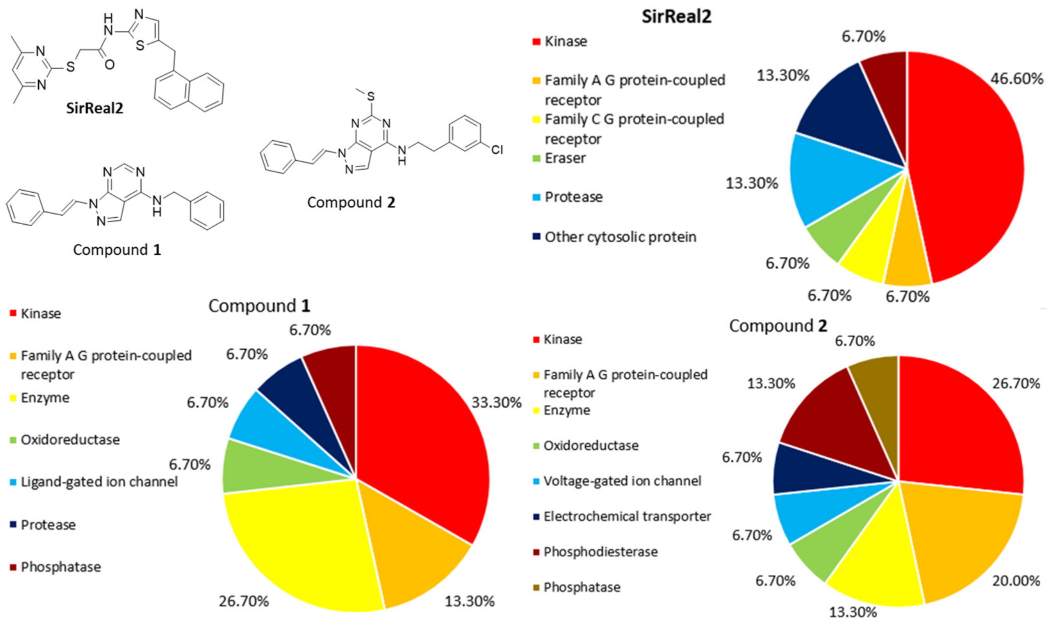

Figure S9. Prediction of putative target preferences displayed by SirReal2 and compounds **1**-**2** via SwissTarget [58].

Table S1. Screening performances according to all the scores available in ICM Pro are shown. A color gradient from yellow (low performances) to green (high performance) was used to highlight the best structures for each scoring function.

Structure	AUC (Egv)	AUC (Edock)	AUC (Egb)	AUC (Ege)	AUC (Egs)
5Y5N	0.804	0.678	0.294	0.315	0.601
4RMH	0.673	0.559	0.409	0.409	0.520
5DY4	0.643	0.535	0.423	0.524	0.532
5DY5	0.642	0.564	0.373	0.641	0.469
4RMG	0.634	0.548	0.520	0.555	0.515
5MAR_	0.592	0.514	0.526	0.639	0.362
5YQL	0.591	0.517	0.507	0.618	0.446
5MAT	0.585	0.495	0.420	0.537	0.462
5YQM	0.576	0.502	0.467	0.558	0.481
4RMJ	0.557	0.484	0.543	0.505	0.344
1J8F	0.551	0.486	0.601	0.608	0.456
5YQO	0.549	0.506	0.579	0.574	0.435
4RMI	0.547	0.476	0.451	0.623	0.519
5D7Q	0.533	0.461	0.549	0.530	0.547
5YQN	0.525	0.494	0.601	0.608	0.506
5D7P	0.518	0.472	0.594	0.591	0.463
5D7O	0.516	0.492	0.675	0.617	0.455
3ZGO	0.516	0.466	0.592	0.604	0.400
3ZGV	0.511	0.469	0.610	0.641	0.386
6QCN	0.511	0.466	0.478	0.536	0.447
5Y0Z	0.497	0.443	0.392	0.608	0.352

Table S2. Prospectical VS hitlist derived by the shared best ranked ChemDiv compounds based on the Edock and Egv lists. The Egv and Edock overall values spanning from -100 to -27 and from -79 to +154, respectively. The best ranked compounds herein reported as **1**,**2**,**3**, **30** and **32** correspond to G779-0661, L929-0391, T158-0512, S787-1020 and L407-0319, respectively.

Compound	Edock	Egb	Ege	Egs	Egv

**1**	-74.1010	-0.0015	-3.1336	-3.9952	-98.1924
**2**	-73.1753	-2.3449	-2.1623	-5.7277	-97.6812
**3**	-73.1105	-0.0372	-1.4511	-4.0467	-98.3821
**4**	-73.0800	-0.0017	-3.7219	-4.2774	-93.4413
**5**	-72.9835	-0.0135	-0.7816	-5.5705	-95.7549
**6**	-71.7816	-0.3475	-0.1681	-4.7204	-98.9049
**7**	-71.5094	-0.0248	-1.3601	-4.0547	-93.7375
**8**	-69.9059	-2.7525	-4.1420	-3.8886	-94.5518
**9**	-69.0341	-1.9179	-1.8940	-4.8107	-93.4752
**10**	-66.9884	-0.0094	-1.6075	-4.1240	-99.7078
**11**	-65.0870	0.0000	0.3325	-5.6524	-95.6800
**12**	-64.6306	-0.0009	-0.6915	-6.4698	-97.4875
**13**	-64.5609	0.0000	-1.3661	-4.6546	-94.0085
**14**	-64.4532	-1.9053	-2.3282	-5.1569	-104.8000
**15**	-64.2745	-0.5489	-2.8789	-3.9479	-94.9102
**16**	-64.2445	-2.5014	-2.7627	-3.3587	-94.7383
**17**	-63.9475	0.0000	-2.4020	-2.7798	-93.3818
**18**	-63.6607	-2.5639	-3.3902	-4.5794	-99.5819
**19**	-63.5323	-0.3831	-1.8376	-3.8880	-101.4200
**20**	-63.3577	-2.2523	-4.1377	-5.0910	-102.2760
**21**	-63.1814	-0.8960	-3.2990	-4.5044	-105.1260
**22**	-62.9697	-2.5862	-3.3027	-4.9437	-103.6320
**23**	-62.7408	-0.0002	-1.3012	-5.0084	-96.5661
**24**	-62.5634	-0.7837	-2.8341	-5.2580	-101.2800
**25**	-62.4619	-0.0389	-1.9369	-4.3631	-97.5730
**26**	-62.4229	-0.1062	-0.5760	-5.2396	-98.4668
**27**	-62.3099	-0.0044	-2.2245	-5.1281	-97.9797
**28**	-62.2316	-0.0005	-4.2195	-4.4760	-94.0403
**29**	-62.0531	-0.0001	-1.6504	-4.3139	-96.7160
**30**	-61.9968	-2.5799	-3.5404	-4.4009	-98.1765
**31**	-61.8770	-0.0637	-0.2563	-4.5305	-95.2331
**32**	-61.7207	-2.8779	-1.5110	-6.1593	-95.9497
**33**	-61.5949	-0.8718	-4.6105	-4.6819	-104.8680
**34**	-61.4346	-0.1157	-0.8091	-5.5982	-97.5079
**35**	-61.3821	-0.8690	-3.2354	-4.0503	-105.1150
**36**	-61.3133	-2.5714	-3.8932	-3.9555	-100.8300
**37**	-61.2720	-2.6280	-3.5496	-4.7674	-102.0800
**38**	-61.1623	-0.0006	-2.2522	-4.0464	-93.9030
**39**	-60.6692	-0.6984	-2.3879	-4.4881	-94.3983
**40**	-60.6456	-0.7940	-3.2206	-3.8874	-101.5290
**41**	-60.6189	-0.0877	-1.8410	-4.3481	-93.7782
**42**	-60.4993	-0.0001	-4.2309	-3.7123	-94.5240
**43**	-60.4117	-1.1882	-2.0536	-4.2117	-98.9525
**44**	-60.3808	-0.5576	-2.0215	-4.4631	-93.9850
**45**	-60.2867	-1.4237	-3.9941	-3.8708	-98.7257

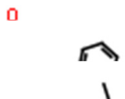

**46**	-60.2196	-2.8228	-2.9219	-6.2219	-95.0871
**47**	-60.0609	0.0000	-2.5361	-3.5206	-93.2596
**48**	-59.8568	-1.5263	-2.2573	-6.2298	-95.2045
**49**	-59.7578	-2.0971	-1.2352	-3.6144	-98.7815
**50**	-59.5333	-0.0016	-0.9065	-5.3786	-108.2460
**51**	-59.4172	-0.6711	-4.6271	-3.9765	-101.1850
**52**	-59.3197	-0.0006	-1.7961	-4.4074	-93.5351
**53**	-59.3128	-0.0028	-0.2272	-4.5865	-94.5272
**54**	-59.2622	-0.6176	-3.0479	-4.6180	-108.2000
**55**	-59.1593	-0.6183	-3.2467	-4.1109	-103.0490
**56**	-59.1300	-0.1482	-0.9154	-4.6027	-94.3369
**57**	-59.0794	-0.9283	-3.2935	-4.3358	-105.0790
**58**	-59.0727	-0.0009	-1.2124	-6.0624	-93.6329
**59**	-58.9055	-0.0415	-2.6879	-4.6307	-103.3830
**60**	-58.8999	-1.1390	-2.5991	-5.5194	-100.0400
**61**	-58.8606	-0.6398	-4.5510	-4.0584	-96.8498
**62**	-58.2566	-0.9527	-4.0778	-4.2850	-96.8092
**63**	-58.1412	-1.4077	-2.2307	-4.9205	-94.1710
**64**	-58.0369	-0.0003	-2.2669	-4.8472	-97.2731
**65**	-57.8591	-1.5607	-2.9456	-6.1299	-95.9226
**66**	-57.6873	-0.2497	-1.6056	-4.1594	-95.0826
**67**	-57.3347	-0.0661	-1.2544	-4.5302	-98.0901
**68**	-57.2832	-2.7845	-2.1933	-6.4634	-99.0789
**69**	-57.2566	-0.0091	-2.4627	-4.3892	-93.8245
**70**	-57.1401	-0.0017	-3.1232	-3.6074	-94.7271
**71**	-57.1012	-0.0006	-3.6977	-3.6625	-95.7957
**72**	-56.7250	-2.3513	-3.1346	-4.4361	-97.6273
**73**	-56.7175	-0.1313	-1.1070	-5.1882	-107.7800
**74**	-56.7174	-0.0090	-2.4388	-4.7531	-93.7740
**75**	-56.6428	-2.1925	-2.0195	-3.3692	-98.1029
**76**	-56.4770	-0.0618	-2.8450	-4.5229	-95.4513
**77**	-56.4366	0.0000	-3.4258	-3.9035	-96.2544
**78**	-56.3899	-1.4357	-5.4366	-4.8446	-97.4825
**79**	-56.3272	-2.0205	-6.1612	-4.0414	-96.3520
**80**	-56.2961	-0.0024	-1.5220	-5.1081	-102.2000
**81**	-56.0894	-0.9316	-3.2638	-4.2895	-93.1568
**82**	-55.9252	-0.0674	-1.6011	-4.7996	-96.9458
**83**	-55.7163	-0.0137	-0.7777	-3.5116	-94.5758
**84**	-55.7091	-0.0049	-2.3269	-4.6630	-93.0870
**85**	-55.6702	-1.5347	-2.6694	-6.4437	-95.6793
**86**	-55.6015	-0.3624	-0.6558	-4.2062	-95.3834
**87**	-55.5642	-2.1875	-2.9041	-5.0136	-94.8598
**88**	-55.3460	-0.0081	-3.1021	-2.8365	-94.6978
**89**	-55.2981	-0.2023	-3.2514	-5.2092	-97.1420
**90**	-55.2835	-2.2083	-3.1451	-5.2238	-96.3453
**91**	-55.2227	-1.4475	-0.6717	-4.1146	-100.5660

**92**	-55.1365	-0.0292	-2.6166	-4.0825	-100.4020
**93**	-55.0291	-0.0119	-2.3920	-3.9357	-95.0055
**94**	-54.9067	-0.7622	-4.3134	-3.9374	-102.6300
**95**	-54.7922	-2.8035	-5.6293	-5.1247	-98.3778
**96**	-54.7778	-0.2550	-1.9831	-4.4438	-97.0222
**97**	-54.6720	-0.0073	-1.4701	-4.6710	-96.5072
**98**	-54.6015	-0.1328	-1.0676	-4.8723	-96.4729
**99**	-54.4797	-0.0001	-4.6508	-4.3936	-93.9290
**100**	-54.4442	-0.6699	-2.3302	-4.7279	-100.2480
**101**	-54.2429	-1.4547	-2.7816	-4.4133	-94.2846
**102**	-54.2413	-0.0849	-3.6666	-5.0271	-102.0420
**103**	-54.1346	-2.6935	-0.1961	-4.5565	-100.3350
**104**	-54.1119	-0.0563	-4.1697	-3.1698	-103.1620
**105**	-53.8940	-0.0755	-1.2928	-4.1627	-94.9840
**106**	-53.8502	-0.0095	-1.3343	-4.1158	-95.3158
**107**	-53.6962	0.0000	-2.9883	-4.2593	-95.4560
**108**	-53.6106	-0.0084	-2.5255	-4.2487	-104.4230
**109**	-53.0678	-0.1815	-1.9526	-5.7253	-99.8734
**110**	-52.9804	-0.0099	-3.0822	-3.1097	-93.3653
**111**	-52.9516	-0.0047	-3.4885	-4.6460	-95.7773
**112**	-52.7471	0.0000	0.6760	-6.2456	-95.8534
**113**	-52.7269	-0.1925	-2.9846	-5.4825	-97.4698
**114**	-52.5380	-0.0298	-2.5814	-4.2068	-96.6420
**115**	-52.5095	-1.1778	-0.5474	-4.6805	-97.6060
**116**	-52.4309	-2.9244	-2.0999	-4.7908	-94.4248
**117**	-52.3048	-0.0006	-2.4517	-4.4511	-96.1636
**118**	-52.2837	0.0000	-1.8656	-4.8776	-94.2555
**119**	-52.0834	-0.1454	-2.6446	-5.0120	-101.6250
**120**	-52.0529	-0.1993	-1.5629	-5.4449	-98.0737
**121**	-52.0324	-0.0202	-2.6802	-3.7517	-97.8603
**122**	-51.9735	-2.3193	-2.1231	-4.7037	-93.0883
**123**	-51.9201	-0.0264	-2.5331	-4.0478	-99.0031
**124**	-51.8823	0.0000	-3.4827	-5.1025	-95.7570
**125**	-51.8631	-1.9610	-7.1271	-3.7703	-96.7123
**126**	-51.8626	-2.5053	-4.5323	-4.2030	-96.5582
**127**	-51.7673	-0.6655	-2.1999	-4.4758	-96.0942
**128**	-51.5975	-0.1582	-1.2830	-4.4468	-99.0986
**129**	-51.4326	0.0000	-0.8297	-4.3733	-96.9515
**130**	-51.3839	-0.3403	-1.3218	-3.9589	-95.6809
**131**	-51.2375	-0.0378	-3.1637	-3.0342	-94.6419
**132**	-51.2301	-0.0038	-1.2090	-4.5928	-99.4314
**133**	-51.2019	-0.0160	-5.1322	-3.8772	-99.6118
**134**	-51.1852	-0.0858	1.2446	-4.4477	-96.7682
**135**	-51.1296	-0.0003	-5.1402	-3.4187	-93.6378
**136**	-51.0505	-0.9464	-2.0487	-3.6821	-95.2863
**137**	-50.9949	-3.9262	-4.5268	-4.9822	-100.8360

**138**	-50.9850	-2.0310	-4.5483	-2.8759	-93.3405
**139**	-50.9300	-2.1170	-2.7666	-5.1867	-94.6087
**140**	-50.8604	-0.0442	-1.7295	-5.0401	-97.4395
**141**	-50.7745	-1.8347	-3.3052	-2.9077	-99.4844
**142**	-50.6867	-0.0159	-5.2116	-3.8444	-102.4420
**143**	-50.5105	-0.1448	-3.5850	-3.3833	-95.5918
**144**	-50.5047	-0.0770	-3.8328	-4.2863	-98.2482

Table S3. List of the docked positioning at the PDB code 5Y5N as obtained for the ChemDiv compounds L407-0319, T158-0512, L929-0391, S787-1020 and G779-0661 by the MOE software, via Induced Fit Method. The related scoring functions are reported (see Materials and Methods for details). Each compound best ranked positioning is numbered in brackets.

Compound	S	E_conf	E_place	E_score1	E_refine	E_score2
**S787-1020** (1)	-12.9629	-56.3911	-28.1496	-6.9868	-6.0236	-12.9629
**S787-1020** (2)	-12.8906	-58.1482	-29.4084	-10.9565	-3.8008	-12.8906
**S787-1020** (3)	-12.7075	-52.3098	-28.3601	-10.6552	-0.3903	-12.7075
**S787-1020** (4)	-12.5217	-49.5478	-33.5206	-8.4583	3.3984	-12.5217
**S787-1020** (5)	-12.3914	-47.3594	-27.6094	-6.4112	21.2888	-12.3914
**S787-1020** (6)	-11.3572	-38.6717	-30.9583	-7.0214	38.3946	-11.3572
**S787-1020** (7)	-11.2510	-42.8353	-33.3823	-7.8193	24.5549	-11.2510
**S787-1020** (8)	-11.1606	-19.9268	-30.0418	-5.2849	43.0576	-11.1606
**S787-1020** (9)	-11.0530	-7.2810	-31.1057	-6.3583	39.6091	-11.0530
**S787-1020** (10)	-10.7224	-43.2943	-15.7638	-5.5554	-0.0271	-10.7224
**G779-0661** (1)	-11.0609	-93.0590	-29.4996	-6.5924	-3.2255	-11.0609
**G779-0661** (2)	-10.8909	-100.1790	-27.5835	-6.2791	-1.4830	-10.8909
**G779-0661** (3)	-10.8036	-91.2914	-22.1520	-8.4753	-7.3028	-10.8036
**G779-0661** (4)	-10.7843	-92.5569	-19.2095	-6.9647	-4.3070	-10.7843
**G779-0661** (5)	-10.7174	-91.0160	-19.5722	-6.2984	-3.9622	-10.7174
**G779-0661** (6)	-10.5314	-94.8143	-22.9798	-6.8813	-8.0070	-10.5314
**G779-0661** (7)	-10.1919	-92.2446	-25.3268	-6.3812	-6.4496	-10.1919
**G779-0661** (8)	-10.1027	-97.9148	-23.9967	-8.3370	-3.0042	-10.1027
**G779-0661** (9)	-9.6690	-71.9892	-18.8026	-7.9855	15.0713	-9.6690
**G779-0661** (10)	-9.4174	-99.1317	-19.8047	-7.2923	-2.0284	-9.4174
**L929-0391** (1)	-11.4816	-13.6089	-25.7211	-6.6640	-9.8897	-11.4816
**L929-0391** (2)	-11.3849	-6.9678	-22.6603	-7.5868	9.4724	-11.3849
**L929-0391** (3)	-11.2309	-8.1932	-38.7532	-11.0587	-6.0178	-11.2309
**L929-0391** (4)	-10.9817	4.4021	-28.3159	-7.2305	3.0133	-10.9817
**L929-0391** (5)	-10.9782	3.5696	-26.7625	-7.3105	13.8290	-10.9782
**L929-0391** (6)	-10.9284	4.6133	-23.8885	-7.7201	8.9119	-10.9284
**L929-0391** (7)	-10.7332	-12.4066	-24.5360	-6.9871	-3.3393	-10.7332

**L929-0391** (8)	-10.6277	-2.8772	-32.5934	-8.3447	9.5693	-10.6277
**L929-0391** (9)	-10.4772	-4.1015	-25.9803	-6.6437	3.8881	-10.4772
**L929-0391** (10)	-10.0302	7.1742	-31.5802	-7.6948	2.0055	-10.0302
**L407-0319** (1)	-10.9991	22.4051	-28.9169	-8.6297	0.2398	-10.9991
**L407-0319** (2)	-10.6003	19.9485	-32.1959	-6.8201	17.6211	-10.6003
**L407-0319** (3)	-10.5481	16.9632	-23.9989	-7.1032	8.9507	-10.5481
**L407-0319** (4)	-10.4156	0.8449	-26.3849	-6.9491	3.5253	-10.4156
**L407-0319** (5)	-10.4034	24.4647	-29.2555	-8.8818	6.7530	-10.4034
**L407-0319** (6)	-10.3856	11.8233	-24.8855	-7.3133	14.6330	-10.3856
**L407-0319** (7)	-10.3253	27.9439	-28.9309	-7.9709	29.5893	-10.3253
**L407-0319** (8)	-10.3247	36.7086	-32.1794	-8.6008	25.6048	-10.3247
**L407-0319** (9)	-10.2434	20.5182	-28.6654	-7.2897	14.9559	-10.2434
**L407-0319** (10)	-10.0754	11.5451	-25.3714	-7.2537	21.5696	-10.0754
**T158-0512** (1)	-11.2678	-84.1918	-38.2289	-10.4326	-17.1116	-11.2678
**T158-0512** (2)	-11.1999	-75.5950	-22.8768	-7.0069	-0.6193	-11.1999
**T158-0512** (3)	-10.9673	-58.5861	-18.6111	-7.7297	10.1871	-10.9673
**T158-0512** (4)	-10.9055	-69.7515	-24.2180	-7.4382	0.6890	-10.9055
**T158-0512** (5)	-10.8869	-66.3196	-24.5560	-7.5278	-7.1103	-10.8869
**T158-0512** (6)	-10.6946	-72.8045	-30.8092	-7.1964	-4.7896	-10.6946
**T158-0512** (7)	-10.6148	-72.1135	-22.0245	-7.6108	1.8093	-10.6148
**T158-0512** (8)	-10.6148	-75.5431	-25.8115	-7.2905	8.2533	-10.6148
**T158-0512** (9)	-10.5834	-62.1496	-33.4858	-9.3133	5.5085	-10.5834
**T158-0512** (10)	-10.5123	-60.4017	-25.0967	-11.0992	37.9273	-10.5123

Table S4. List of the docked positioning at the PDB code 5Y5N as obtained for the in-house pyrazolopyrimidine derivatives **1**-**5** and for **AGK2** by the MOE software, via Induced Fit Method. The related scoring functions are reported (see Materials and Methods for details). Each compound (Comp.) best ranked positioning is numbered in brackets.

**Comp.**	**SIRT2 activity, % inhibition (at 150μM)**	**S**	**E_conf**	**E_place**	**E_score1**	**E_refine**	**E_score2**
**AGK2** (1)		-13.3422	42.0134	-24.8074	-4.9204	-8.0481	-13.3422
**AGK2** (2)		-13.1782	32.6195	-24.9241	-5.6795	-12.1494	-13.1782
**AGK2** (3)		-13.0759	44.7573	-24.4907	-7.1738	7.9173	-13.0759
**AGK2** (4)		-12.7270	56.8438	-22.2558	-5.2599	11.4438	-12.7270
**AGK2** (5)		-12.6897	34.5196	-19.6450	-5.5092	4.5140	-12.6897
**AGK2** (6)		-12.5827	49.8805	-25.7144	-4.7816	5.9234	-12.5827
**AGK2** (7)		-12.3439	84.1997	-28.3850	-6.9059	20.8000	-12.3439
**AGK2** (8)	97±10	-12.0308	91.3584	-21.4676	-4.7684	73.4215	-12.0308
**AGK2** (9)		-11.3564	30.9502	-17.1473	-5.7395	-11.3315	-11.3564
**AGK2** (10)		-11.0339	34.0729	-15.3933	-5.6749	-8.0045	-11.0339
Comp. **1** (1)		-10.1235	32.7543	-20.9708	-8.0659	-9.2104	-10.1235
Comp. **1** (2)		-9.4463	25.1215	-20.3997	-7.8768	-11.5007	-9.4463
Comp. **1** (3)		-9.1678	23.2598	-17.0396	-8.9267	-9.1595	-9.1678
Comp. **1** (4)		-8.8686	18.7720	-22.2926	-8.3693	-12.4879	-8.8686

Comp. **1** (5)		-8.5445	21.2742	-18.5652	-9.3955	-5.7665	-8.5445
Comp. **1** (6)	81.2±7.3	-8.4330	21.0701	-17.6653	-8.0572	-14.1552	-8.4330
Comp. **1** (7)		-8.3396	32.8019	-27.8831	-8.8722	12.4342	-8.3396
Comp. **1** (8)		-7.6088	22.3001	-17.8534	-8.0659	-9.3113	-7.6088
Comp. **1** (9)		-7.5627	23.6958	-13.9132	-10.3548	-10.1522	-7.5627
Comp. **1** (10)		-7.2794	22.3064	-15.0052	-8.4840	-9.2212	-7.2794
Comp. **2** (1)		-10.6011	78.4224	-23.4699	-5.1424	46.1146	-10.6011
Comp. **2** (2)		-10.4132	45.4204	-10.2007	-5.1029	11.4587	-10.4132
Comp. **2** (3)		-10.1726	29.4811	-18.7644	-7.7152	4.3011	-10.1726
Comp. **2** (4)		-10.1081	29.1231	-10.5693	-5.2501	6.0333	-10.1081
Comp. **2** (5)		-10.0823	27.7032	-13.7296	-6.6773	7.4934	-10.0823
Comp. **2** (6)		-10.0200	43.8283	-15.6982	-7.8617	15.4735	-10.0200
Comp. **2** (7)	79.9±5.7	-10.0159	27.7095	-16.6155	-5.5088	8.2891	-10.0159
Comp. **2** (8)		-9.6562	15.5165	-17.0470	-9.1955	4.8109	-9.6562
Comp. **2** (9)		-9.6085	35.7236	-11.2675	-6.6172	18.2979	-9.6085
Comp. **2** (10)		-9.4280	18.1683	-22.3381	-9.3905	21.9861	-9.4280
Comp. **3** (1)		-8.9098	32.3784	-18.8613	-10.0137	-4.1164	-8.9098
Comp. **3** (2)		-8.8555	34.7034	-19.6096	-9.3624	-8.7683	-8.8555
Comp. **3** (3)		-8.5589	32.5533	-17.0152	-9.1667	-9.1626	-8.5589
Comp. **3** (4)		-8.4621	40.5428	-22.9374	-8.8308	14.8686	-8.4621
Comp. **3** (5)	51.1±15.2	-8.1030	25.4036	-18.6497	-8.5754	-13.4334	-8.1030
Comp. **3** (6)		-8.0371	37.0049	-17.9247	-9.1068	-7.0459	-8.0371
Comp. **3** (7)		-7.9924	54.3745	-17.6777	-8.9269	11.6464	-7.9924
Comp. **3** (8)		-7.8812	42.2742	-22.0742	-8.5052	10.1671	-7.8812
Comp. **3** (9)		-7.8392	44.4200	-22.1329	-9.4621	13.3254	-7.8392
Comp. **3** (10)		-7.4396	57.1753	-19.1514	-9.5890	-4.0853	-7.4396
Comp. **4** (1)		-10.2455	50.4786	-28.9652	-9.0331	0.6912	-10.2455
Comp. **4** (2)		-9.8068	50.9603	-22.3115	-7.5869	23.6100	-9.8068
Comp. **4** (3)		-9.7042	64.1719	-30.9991	-8.2975	10.7053	-9.7042
Comp. **4** (4)		-9.5734	41.1254	-23.5727	-8.0481	24.9652	-9.5734
Comp. **4** (5)	35.7±2.5	-9.3790	54.8781	-22.5588	-7.7471	15.8612	-9.3790
Comp. **4** (6)		-9.0861	45.4645	-23.9362	-8.5249	32.7465	-9.0861
Comp. **4** (7)		-8.4322	29.3551	-20.6842	-8.6190	-5.9255	-8.4322
Comp. **4** (8)		-8.1193	32.5835	-20.8686	-9.4813	8.3224	-8.1193
Comp. **4** (9)		-8.0110	65.7885	-19.2858	-8.1719	31.8408	-8.0110
Comp. **4** (10)		-7.7538	33.4744	-20.7952	-8.2916	17.8685	-7.7538
Comp. **5** (1)		-8.3733	14.1298	-21.5673	-10.2491	-5.6128	-8.3733
Comp. **5** (2)		-8.0763	11.6770	-22.4232	-9.7738	-10.3065	-8.0763
Comp. **5** (3)		-8.0670	21.7656	-23.8852	-9.3472	20.0158	-8.0670
Comp. **5** (4)		-7.9753	11.7914	-23.5345	-9.7690	-9.6618	-7.9753
Comp. **5** (5)		-7.9118	16.5507	-21.9831	-10.4226	-9.4711	-7.9118
Comp. **5** (6)	31.4	-7.7647	29.9488	-26.6388	-9.4354	-5.7638	-7.7647
Comp. **5** (7)		-7.7461	24.2195	-21.1790	-9.5222	-2.2892	-7.7461
Comp. **5** (8)		-7.5208	25.7669	-22.3904	-10.0996	2.3178	-7.5208
Comp. **5** (9)		-7.3605	31.3796	-22.4805	-10.5198	14.2673	-7.3605
Comp. **5** (10)		-7.1799	21.2991	-26.7483	-9.4834	-5.3364	-7.1799

Table S5. Calculated features as suggested by the Lipinski's and Veber's rules and by other pharmacokinetic (PK) properties, referred to the novel compounds (Comp.) **1–5**. The same prediction for the reference inhibitor SirReal2 has been listed. The predicted values for the effective pyrazolo-pyrimidine **1** as ameliorated druglike compound, with respect to SirReal2 (in grey), are highlighted in light cyan. Reliability index values for a number of descriptors are shown as R.I. (values higher than 0.30 are ranked as reliable by the software).

Comp.	MW[a] (g/mol)	HBA[b]	HBD[c]	N. rot. bonds[d]	LogP[e]	TPSA[f]	HIA (%)[g]	Vd (l/kg)[h]	%PPB[i] (R.I. ≥ 0.60)	LogKa[l] HSA (R.I. ≥ 0.40)	%F (oral)[m] 50mg
**1**	327.38	3	1	5	2.88	55.63	100	2.7	98.57	4.77	96.6
**2**	421.95	3	1	7	4.21	80.93	100	4.3	99.97	5.04	38.0
**3**	309.37	4	1	7	2.00	64.86	100	1.9	95.55	4.09	99.3
**4**	319.40	3	0	3	2.87	46.84	100	3.5	96.54	4.30	91.6
**5**	277.32	3	1	4	2.16	55.63	100	2.1	96.24	3.79	98.4
**SirReal2**	420.55	4	1	7	2.65	121.31	100	2.5	98.71	4.69	21.9

[a] Molecular weight; [b] Number of H-bond acceptors; [c] Number of H-bond donors; [d] Number of rotatable bonds; [e] Logarithmic ratio of the octanol–water partitioning coefficient; [f] Topological polar surface area; [g] HIA represents the human intestinal absorption, expressed as percentage of the molecule able to pass through the intestinal membrane; [h] Prediction of Volume of Distribution (Vd) of the compound in the body; [i] Ligand affinity toward human serum albumin (HSA); [m] Percentage oral bioavailability

Table S6. Calculated ADMET descriptors related to metabolism, excretion and toxicity properties, referred to the novel compounds (Comp.) **1–5** and to SirReal2. The pyrazolo-pyrimidine **1** and SirReal2 are highlighted in light cyan and grey, respectively.

Comp.	Endocrine System Disruption[a] (R.I. ≥ 0.30)		CYP3A4[b] (R.I. ≥ 0.30)		LD_{50} (mg/kg)[c] Mouse oral (R.I. ≥ 0.30)	hERG inhibitor [d] (R.I = 0.20)	PAINS (Pan Assay Interference structures)
	LogRBA > -3	LogRBA > 0	Inhibitor (IC_{50} < 10µM)	Substrate			
**1**	**0.58**	**0.03**	0.76	0.87	1000	0.15	0 ALERT
**2**	0.52	0.02	0.79	0.95	1000	0.24	0 ALERT
**3**	0.15	0.00	0.47	0.63	930	0.17	0 ALERT
**4**	0.41	0.05	0.36	0.74	650	0.30	0 ALERT

**5**	0.09	0.00	0.44	0.58	540	0.09	0 ALERT
**SirReal2**	0.34	0.09	0.38	0.97	1100	0.15	0 ALERT

[a] RBA represents the relative binding affinity with respect to that of estradiol. Compounds showing LogRBA > 0 are classified as strong estrogen binders, while those showing LogRBA < -3 are considered as non-binders; [b] Prediction of the ligand inhibitor/substrate behavior towards cytochrome CYP3A4 [c] Acute toxicity (LD_{50}) for mice after oral administration; [d] Probability of human ether-á-go-go-related gene (hERG) channel inhibition at clinically relevant concentrations (K_i<10 µM).
